# A Systematic Review on Attenuation of PCSK9 in Relation to Atherogenesis Biomarkers Associated with Natural Products or Plant Bioactive Compounds in In Vitro Studies: A Critique on the Quality and Imprecision of Studies

**DOI:** 10.3390/ijerph191912878

**Published:** 2022-10-08

**Authors:** Rahayu Zulkapli, Mohd Yusmiaidil Putera Mohd Yusof, Suhaila Abd Muid, Seok Mui Wang, Al’Aina Yuhainis Firus Khan, Hapizah Nawawi

**Affiliations:** 1 Institute of Pathology, Laboratory and Forensic Medicine (I-PPerForM), Universiti Teknologi MARA (UiTM), Sungai Buloh Campus, Jalan Hospital, Sungai Buloh 47000, Selangor, Malaysia; 2 Faculty of Medicine, Universiti Teknologi MARA (UiTM), Sungai Buloh Campus, Jalan Hospital, Sungai Buloh 47000, Selangor, Malaysia; 3 Faculty of Dentistry, Universiti Teknologi MARA (UiTM), Sungai Buloh Campus, Jalan Hospital, Sungai Buloh 47000, Selangor, Malaysia

**Keywords:** PCSK inhibitor, PCSK9, endothelial cells, natural products, atherogenesis, atherosclerosis

## Abstract

A systematic review was performed to identify all the related publications describing PCSK9 and atherogenesis biomarkers attenuation associated with a natural product and plant bioactive compounds in in vitro studies. This review emphasized the imprecision and quality of the included research rather than the detailed reporting of the results. Literature searches were conducted in Scopus, PubMed, and Science Direct from 2003 until 2021, following the Cochrane handbook. The screening of titles, abstracts, and full papers was performed by two independent reviewers, followed by data extraction and validity. Study quality and validity were assessed using the Imprecision Tool, Model, and Marker Validity Assessment that has been developed for basic science studies. A total of 403 articles were identified and 31 of those that met the inclusion criteria were selected. 13 different atherogenesis biomarkers in relation to PCSK9 were found, and the most studied biomarkers are LDLR, SREBP, and HNF1α. In terms of quality, our review suggests that the basic science study in investigating atherogenesis biomarkers is deficient in terms of imprecision and validity.

## 1. Introduction

Systematic reviews in the context of basic research are uncommon. However, despite the rareness, there were systematic reviews of in vitro studies [1,2,3,4]. Systematic reviews for basic science provide the same benefits as those conducted for preclinical animal studies: to statistically combine the results of numerous related studies to provide more reliable results on which decisions can be made and evidence gaps are identified. Basic science can be translated into clinical practice based on solid evidence, and basic research validation is improved by identifying results within multiple model systems [5].

The proprotein convertase subtilisin/Kexin type 9 (PCSK9) has gained attention as a potential therapeutic target for lowering cholesterol levels, especially in homozygous familial hypercholesterolemia (FH)/high-risk and/or category patients who do not reach the low-density lipoprotein (LDL) target, a major risk factor for cardiovascular diseases [6,7,8]. The discovery of the 9th or the last member of the protein convertase family known as PCSK9 was reported in 2003 by Nabil Seidah [9]. Until it was discovered, there were only two known genes (*LDL-R and ApoB*) related to FH in humans [10]. The classical method of action involves PCSK9 protein chaperoning the low-density lipoprotein receptor (LDLR) to intracellular degradative organelles, hence accelerating its degradation [11]. The consequent reduction in surface LDLR impedes LDL clearance, yielding an increase in plasma LDL cholesterol (LDL-C). The discovery of PCSK9 took a sharp turn in the lipid field with PCSK9 inhibitors becoming an undeniable therapeutic reality. Mice and humans without functional PCSK9 appear healthy [12,13], and it seems that therapeutic inhibition of PCSK9 unlikely would have any serious adverse effects. This makes PCSK9 a very promising potential therapeutic target for dyslipidemia therapy.

Currently, numerous prospective medications that inhibit the PCSK9 pathway have entered preclinical or early phase clinical trials, and the FDA has approved two of these treatments (evolocumab and alirocumab) [14,15]. According to preclinical research, PCSK9 has pleiotropic effects beyond regulating plasma LDL-C levels and may be a crucial factor in the pathogenesis of atherosclerosis [16,17]. The PCSK9 inhibition attenuates atherosclerosis progression and lowers the risk for acute cardiovascular events [6,18]. PCSK9 inhibition may be best achieved by identifying and developing small compounds that may be taken orally and have anti-PCSK9 action. The history of pharmacology has offered compelling evidence on the significance of identifying naturally occurring substances with potential therapeutic actions, and the in vitro studies have provided persuasive evidence of the relevance through molecular mechanisms [19]. The atherogenic inhibition by the natural products in in vitro studies was conducted by measuring the expression of the inflammatory, adhesion molecules, oxidative stress, endothelial nitric oxide synthase (eNOS), and nuclear factor-κB (NF-kB) biomarkers [20,21,22].

Therefore, this review aimed to gather, compare and critique the imprecision and quality of the in vitro research that is published on bioactive compounds or natural-product-derived PCSK9 inhibitors involving PCSK9 and atherogenic biomarkers inhibition rather than the detailed reporting of the results evidence.

## 2. Methods

The literature search and systematic review methods adhered to the Cochrane Collaboration guidance [23] to reduce the risk of bias and error. This review allows the Preferred Reporting Items for Systematic Reviews (PRISMA) guidelines (Appendix A) [24].

### 2.1. Definitions

PCSK9 inhibition was defined as the hindrance of PCSK9 molecule binding to e LDLR, so that the LDLR degradation can be prevented, thus increasing LDLR being recycled to the surface of hepatocytes for LDLC uptake, and reducing blood LDLC level. Atherogenesis biomarkers are either protein or gene expression that was affected by atherosclerosis. Natural products were defined as substances or chemicals produced by plants. Plant bioactive compounds referred to a type of chemical found in small amounts in plants.

### 2.2. Search Criteria

Electronic literature searches in the Scopus, PubMed, and Science Direct databases was conducted between 2003 and 2021. The starting year is following the year of *PCSK9 gene* discovery as the third gene linked to autosomal dominant hypercholesterolemia [25]. Search strategies are presented in Appendix B. The selected databases were searched on 27 August 2021 up to 30 August 2021. The publications found using the keyword combinations of ‘Proprotein Convertase Subtilisin Kexin 9 Inhibitor* or PCSK inhibitor*’, ‘cell*’, ‘endothelial cell*’ were included. The clinical, diagnostic, or prognostic outcomes were excluded from the review. The time filter used was from 2003 until 2021 to limit the years of publication search.

### 2.3. Inclusion and Exclusion Criteria

The included studies were the original publications of biomarker expression, either protein and/or gene expression of *PCSK9*, and atherogenesis in in vitro studies. The PCSK9 biomarkers were specifically selected and included. The studies of atherogenesis biomarkers without PCSK9 were excluded. The significance and relevance of the selected literature were evaluated based on their content and type of publication. The studies were excluded if (i) the study used other types of PCSK such as PCSK1 or PCSK8, (ii) the study used PCSK9 to observe effects other than lipid-lowering in other diseases, (iii) human subjects or animals were involved, (iv) they were not written in the English language; and (v) the articles were reviews, commentaries, editorials, unpublished manuscripts, or conference abstracts. The non-English research articles, conference proceedings, abstracts, book chapters, and commentaries were not included. All review articles that included clinical, diagnostic, or prognostic outcomes were excluded.

### 2.4. Study Identification and Selection

After identifying articles in the databases mentioned above, these articles were imported into EndNote X20software (Thompson Reuters, Philadelphia, PA, USA), and duplicate articles were removed. The eligibility criteria was used to conduct the first-level screening of articles using titles and abstracts. Full-text articles were then accessed to determine eligible articles to include in the review. A data extraction form was performed to extract study characteristics, including author(s), year of publication, cell lines used, tested plant bioactive compound (PBC) or natural product (NP), biomarkers measured, and expression at protein and gene levels. The titles and abstracts were independently screened by two authors (R.Z. and A.Y.F.K.).

### 2.5. Data Synthesis

A summary of all the included studies was compiled. The data were sorted according to the cell lines, treatment, and the expression of protein and gene levels. The article that discussed more than one cell line or NP/PBC will be separated into different studies (Table 1). Results were presented alongside overall judgments for concerns regarding the validity and imprecision of the result. The data were extracted by a single review author (R.Z.). To ensure accuracy, another review author (A.Y.F.K.) went through the data independently, and any discrepancies were resolved by the third review author (H.N.).

### 2.6. Quality Assessment

The current review emphasized the imprecision and quality of the included research rather than the detailed reporting of the results.

The only risk of bias for non-clinical research is the SYRCLE checklist [26]. In the SYRCLE checklist, the judgment of the domains is either unclear (UNR) or not applicable (NA). SYRCLE is based on the risk of bias tools developed for randomized controlled clinical trials [24]. However, we found that these tools were not appropriate for the design of basic scientific studies. The SYRCLE signaling questions are not relevant to basic science studies, they do not use a language that is meaningful to laboratory scientists, and they do not critique all issues pertinent to the biases of fundamental research. No formal randomization or allocation concealment or blinding is used in laboratory-based research. In addition, every effort is taken to ensure that the experiment and controls are treated equally throughout the study.

Thus, the validity will follow an “Imprecision Tool and Assessment” (Appendix C), “Model Validity Assessment” (Appendix D), and “Marker Validity Assessment” (Appendix E) established by Collins et al. [1] to judge the choice and validation in basic science studies. In “Imprecision Tool and Assessment”, the determination involves a minimum requirement for low risk was that the authors reported technical repeats, interassay repeats, and variability.

## 3. Results

### 3.1. Literature Searches and Inclusion Assessment

**Figure 1:**
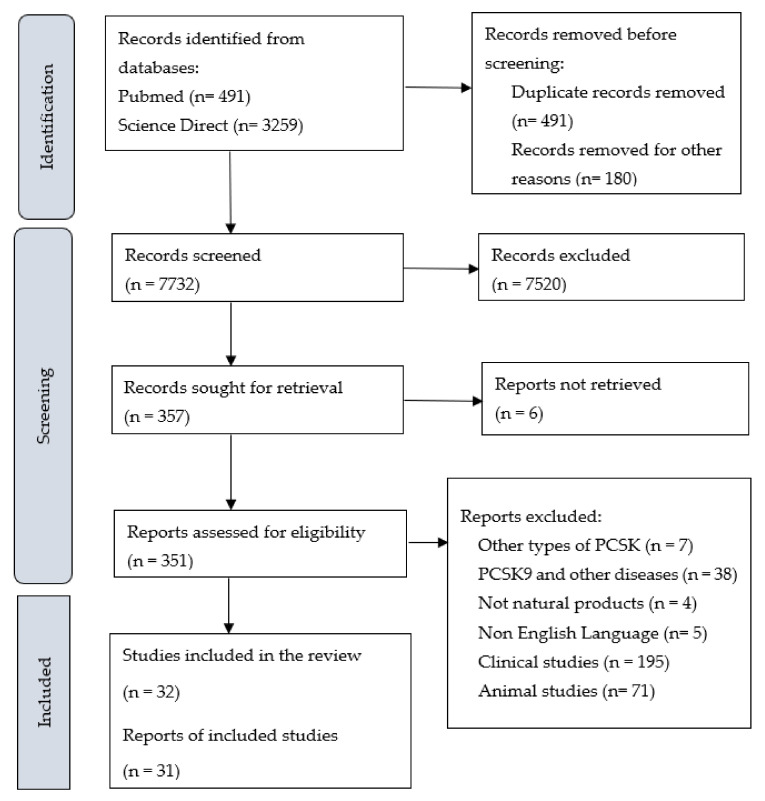
PRISMA Flowchart of Studies.

A summary of the identification and selection of studies for inclusion in this review is presented in Figure 1, in accordance with the PRISMA statement [24]. Literature searches of electronic databases retrieved 8403 research articles. After the duplicated research articles were removed, 8057 titles/abstracts were screened, and 6791 research articles were excluded as having no relevance to the review. Full research articles of 537 potentially relevant references were selected for further examination. Of these, 505 research articles were excluded after reading the entire article; the reasons for exclusion are provided in Figure 1. Thirty-one publications met the inclusion criteria.

### 3.2. PCSK9 in Relation to Atherogenesis Biomarkers

#### 3.2.1. In Vitro Models

Five different cell lines were identified in the in vitro studies that measured the PCSK9 expression. One study reported PCSK9 attenuation in Human Umbilical Vein Endothelial Cells (*HUVEC*), two in Human Hepatocytes (*Huh 7*), twenty-seven in Human hepatoma (*HepG2*), and one in JLM3 (hepatocellular carcinoma cells) (Table 1). Most studies used hepatocytes cell lines, in accordance with the fact that PCSK9 is highly expressed in the liver [58]. Apart from that, PCSK9 is also present in the kidneys, intestines, brain, and blood vessels [59].

Four in vitro models were identified in all selected studies; (i) Oxidized LDL (Ox-LDL) stimulated cells, (ii) Lipopolysaccharides (LPS) stimulated cells, (iii) Lipoprotein-depleted serum (LDPS) cell growth medium, and (iv) Delipidated-serum (DLPS) cell growth medium. Most of the in vitro research selected in the studies used the LPDS model, and the Ox-LDL model was the least used.

#### 3.2.2. Protein and Gene Expression of PCSK9 In Vitro Models

Using systematic review methodology, we identified thirty-two studies describing PCSK9 expression in relation to thirteen different biomarkers studied in human cells line in terms of protein and gene expression, which were treated by different natural products or plant bioactive compounds. All the natural products or compounds in the selected studies possessed downregulated effects of PCSK9 except for red yeast rice (monacolin K) (Table 1).

##### PCSK9 in Relation to LDLR, SREBP, and HNF1α Biomarkers

Twenty-five studies on PCSK9 measured the LDLR expression. From that, all studies showed the inverse relationship between PCSK9 and LDLR levels. However, six studies reported “not significantly changed/unaffected/no changed” in LDLR gene expression even though in the protein expression, it was highly expressed. Second, thirteen studies reported the PCSK9 and SREBP (Sterol regulatory element-binding proteins). From that, seven studies reported the downregulation of SREBP together with PCSK9. Contrarily, two studies reported that SREBP was upregulated when PCSK9 was downregulated, and the other four studies reported “not significantly changed/not affected” on SREBP when PCSK9 was downregulated. Ten studies discussed the HNF1α biomarker in relation to PCKS9; 8 were downregulated with PCSK9 suppression, 1 was upregulated and 1 was not affected by PCSK9 downregulation (Table 1).

These biomarkers were the least biomarkers investigated in the included articles. Four studies investigated the 3-Hydroxy-3-Methylglutaryl-CoA Reductase (HMGCR) biomarkers; only one study reported the protein and gene expression of HMGCR, while the other studies only reported on the mRNA expression. Three studies reported a direct relationship between HMGCR and PCSK9 mRNA, while forkhead box O3 (FoxO3) biomarkers were upregulated in all four studies. The peroxisome proliferator activated Receptor Gamma (PPARg) protein and gene expression was investigated in two studies, and it was unaffected in both. The inverse relationship between PPARg and PCSK9 gene expression was discovered. Lectin-like oxLDL 1 (LOX-1), NADPH Oxidase 4 (NOX-4), adhesion, and inflammatory biomarkers were only reported in one study included. The biomarkers were downregulated only when PCSK9 biomarkers were downregulated. While for fas cell surface death receptor (FAS), only the gene expression was reported, and they were downregulated when PCSK9 was downregulated (Table 1).

#### 3.2.3. Imprecision and Validity Analysis

**Figure 2:**
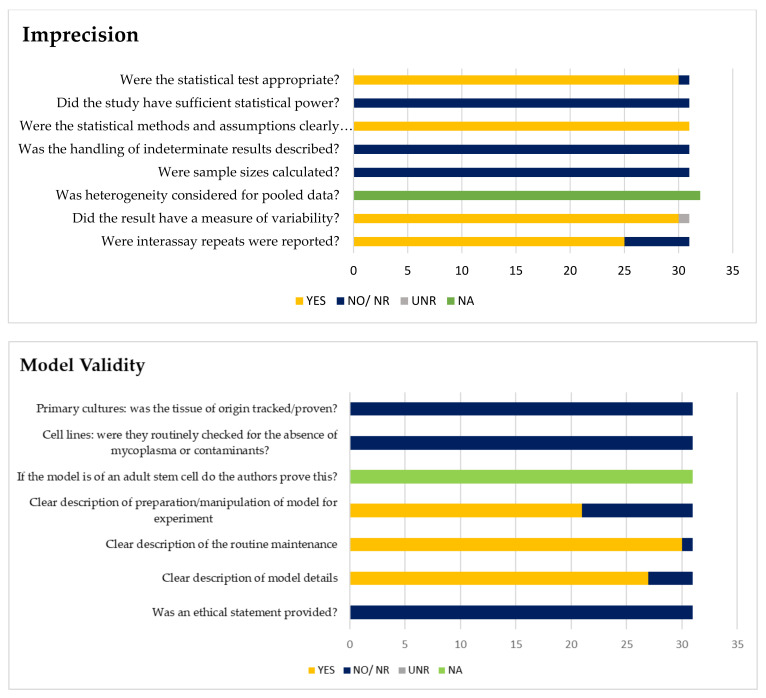
Assessments of imprecision and model validity. Yellow bars = number of studies for judgments of ‘yes’. Dark blue bars = number of studies for judgements of ‘no’ or ‘not reported’. Light grey bars = number of studies ‘unclear (UNR)’ for question (unclear for imprecision). Green bars = number of studies for judgements of ‘not applicable (NA)’.

The imprecision tool for basic science studies was created with the purpose of judging how well the authors reported sample size, statistical methodology, and variability (2). The minimum requirement for low risk is for the authors to have well-reported technical and inter-assay repeats as well as variability. Imprecision Tool Assessment (Figure 2) regarded twenty-seven studies (84%) as ‘low concern’ with low ‘technical reporting and statistical rating’, but the sample size rating was unclear. Another seven studies (22%) were regarded as unclear in all domains during the Imprecision Tool Assessment (Table 2, Figure 2). The imprecision of the included articles was evaluated to be unclear in overall rating when: (1) they scored unclear more in one imprecision domain, (2) the number of technical repeats was not mentioned in the article, and (3) the statistical test rating was reported as unclear because no analysis was reported on the comparison.

A model validity tool performed in basic science is to judge how well the authors reported the details and validity of the model used in the research. Assessment of model validity (Figure 2) indicated that most of the studies (66%) were judged to be valid, ten studies (31%) were unclear, and one was considered to be ‘high concern’; the main reasons lie in the ‘no reported’ model for the experiment.

The marker validity analysis focuses on the most studied biomarkers in the included articles (PCSK9 in relation to LDLR, SREBP, and HNF1α). Analysis of the marker validity for PCSK9 showed eighteen studies (56%) scored ‘low’, while the other fourteen studies were judged to be ‘unclear’ (44%). For LDLR, thirteen studies (52%) were evaluated as ‘low’, and eleven studies were ‘unclear’ (44%). One reported ‘high’ due to the absence of positive and negative control. While SREBP and HNF1α biomarkers were judged to be ‘unclear’ in the majority (92% and 80%) of the included studies for marker validity due to the absence of positive control (Table 2).

## 4. Discussion

Both the mRNA and protein levels of gene expression are controlled by on/off switches and fine-tuned regulation [60]. There has been a flurry of research into the connection between mRNA and protein levels across genes, with sometimes contradicting findings [58]. In yeast, the amount of mRNA present can be used as a reliable predictor of the amount of protein present [59]. On the other hand, in mammalian cells, the association has been demonstrated to be much lower and varies considerably depending on the cell type and state. For cells that have been exposed to a stimulus, the situation gets even more complex. When mammalian cells were exposed to protein misfolding stress, the link between protein and mRNA quantities was broken down, and substantial regulation occurred at both the mRNA and protein levels [61]. Thus, it is crucial to evaluate the quality of the research conducted on the biomarkers specifically in atherogenesis as small changes to the protein and mRNA levels affected the outcome.

To the best of our knowledge, this is the first systematic review that describes the PCSK9 in relation to atherogenesis biomarkers that emphasized the imprecision and quality of the research. A gain-of-function mutation in the PCSK9 gene was found to cause FH [62]. The inhibition of PCSK9 attenuates atherosclerosis progression and reduces the risk for acute cardiovascular events [6,18].

The imprecision analysis, model validity, and marker validity have been performed following the basic science study (2). However, some of the exclusion has been made to suit this study. The sample size rating or evaluation included in the imprecision assessment is not relevant to cell studies as the calculation of sample size is unnecessary before conducting the experiment. In cell studies, triplicates were considered enough when the variation was small. This is agreeable with the majority of the selected and evaluated publications that used technical triplicates in their experiment. Thus, the exclusion of sample size rating is appropriate for the overall imprecision score evaluation. In addition, observer variability (technical reporting domain) also is irrelevant to cell studies research as it requires the paper to report whether the experiment gives the same result when repeated. None of the articles reported on the consistency of the results. Statistical analysis is common and good enough in cell studies to observe variation and consistency. Thus, the observer variability was excluded for the overall score of technical reporting. Other than that, overall, none of the manuscripts describes the routine maintenance of the model (domain four) nor the routine checking for the absence of mycoplasma or contaminants (domain seven). It was a crucial practice and routine in cell culture studies; however, it was rarely reported in the manuscript. The experiment’s success is the actual indicator that the routine was performed. Thus, it was unnecessary to report on that. Therefore, the overall rating was made by excluding the score in domains four and seven. The paper that was regarded as ‘high concern’ or ‘high risk’ is the paper that gave no, not applicable, and not reported for all domains 1 to 9.

All the natural products or compounds in the included studies showed the downregulation of PCSK9 except for red yeast rice (monacolin K). Red yeast rice reported the upregulation of PCSK9 upon treatment with HepG2 (24 h). All included studies showed the inverse relationship between PCSK9 and LDLR levels. This supports the theory that PCSK9-bound-LDLR causes the increase in LDLR degradation that impedes LDLC lowering of PCSK9 by direct binding to the epidermal growth factor repeat A (EGF-A) of the LDLR and shuttling the LDLR from the endosomes to the lysosomes for degradation [63].

SREBP controls the genes involved in fatty acid production (SREBP-1c) and cholesterol metabolism, principally regulating PCSK9 at the transcriptional level (SREBP-2) [64]. The PCSK9 gene minimal promoter region contains a sterol regulatory element (SRE) [65]. Nuclear SREBP expression significantly increases PCSK9 promoter activity, and PCSK9 expression can be controlled by nutritional status via a mechanism involving SREBP-1c [66]. For SREBP, the relationship between PCSK9 and SREBP was contradicted in the included studies; (i) SREBP was upregulated when the PCSK9 was downregulated, and (ii) SREBP “not significantly changed/not affected” when PCSK9 was downregulated. The marker validity was reported as ‘unclear’ for the articles that reported “SREBP was reported not significantly changed nor affected’. Besides SREBP2, HNF1α is a critical transcription factor that regulates PCSK9 gene transcription [65]. Most of the studies showed the downregulation of HNF1α with PCSK9 suppression aggregable with the HNF1α function that promotes PCSK9 transcription by binding with the HNF1 motif, which is located upstream of SRE1 in the PCSK9 promoter [67]. Despite the consensus on the outcome of SREBP and HNF1α in relation to PCSK9, the majority of marker analyses for both were regarded as ‘unclear’ due to the absence of positive control. Very few studies (8% and 20%) reported the positive control of SREBP AND HNF1α biomarkers. The reporting of positive controls should be fundamental in basic science study allowing researchers to validate the outcome of their research.

## 5. Conclusions

Cell lines have long been regarded as a valuable resource for basic research as well as pre-clinical studies. Living cells can be used to investigate the functional significance of genetic products such as mRNA, miRNA, and proteins, and cell lines are a valuable research resource. Studying cell lines is also important in investigating a particular medicine’s detailed mechanism or pathway. Even though selection pressures can compromise the predictive value of cell lines during the formation and long-term passaging processes, a significant advantage of cell lines is that examinations can be conducted with high throughput and at a relatively low cost.

Using a systematic review, the relation of PCSK9 with thirteen different biomarkers in different cell lines has been identified. Despite the exclusion of some criteria domain in the validity and imprecision of the included research, the quality of some studies is still questionable. This might be caused by several factors, especially the cost for basic research to be precise and valid. Improvements are still needed in evaluating the validity and imprecision of basic science studies. The establishment of imprecision and validity for a different scope of basic research, particularly in vitro studies, is crucial as it will allow more rapid development of new alternative treatments.

## Figures and Tables

**Table 1:** Summary of biomarkers expression of selected studies.

Cell Lines	Study ID	Natural Product/ Plant Bioactive Compound	Biomarkers	Expression at Effective Concentration	
				Proteins	Genes
*HUVEC*	Wang 2019 [27]	Ginkgolide B **	PCSK9	Downregulated	Downregulated
			LDLR	Upregulated	Upregulated
			ICAM-1	Downregulated	Downregulated
			VCAM-1	Downregulated	Downregulated
			SREBP2	Downregulated	Downregulated
			IL-1α	Downregulated	Downregulated
			IL-1β	Downregulated	Downregulated
			IL-6	Downregulated	Downregulated
			MCP-1	Downregulated	Downregulated
			CXCL-1	Downregulated	Downregulated
			CXCL-2	Downregulated	Downregulated
			NOX-4	Downregulated	Downregulated
			LOX-1	Downregulated	Downregulated
*Huh7*	Mbikay 2014 [28]	Quercetin-3-glucoside **	PCSK9	Downregulated	Downregulated
			LDLR	Upregulated	Upregulated
			SREBP2	Not reported	Not affected
	Wang 2020 [29]	Ascorbic acid **	PCSK9	Downregulated	Downregulated
			LDLR	Upregulated	Upregulated
			PPARg	Not affected	Not affected
			FoxO3a	Upregulated	Upregulated
*LO2*	Jing 2019 [30]	Resveratrol **	PCSK9	Downregulated	Downregulated
			LDLR	Upregulated	Upregulated
			SREBP 1c	Downregulated	Downregulated
*HepG2*	Aggrey 2019 [31]	3R3,14-dihydroangustoline **	PCSK9	Downregulated	Not reported
			LDLR	Upregulated	Not reported
	Ahn 2019 [32]	Erybraedin D **	PCSK9	Downregulated	Downregulated
	Cameron 2008 [33]	Berberine **	PCSK9	Downregulated	Downregulated
	Chae 2018 [34]	Saucinone **	PCSK9	Not reported	Downregulated
			LDLR	Not reported	Upregulated
	Chen 2016 [35]	Tanshinone IIA **	PCSK9	Downregulated	Downregulated
			LDLR	Upregulated	NSC
	Choi 2017 [36]	*Allium fistulosum* L. *	PCSK9	Downregulated	Downregulated
			LDLR	Downregulated	Downregulated
			SREBP2	Downregulated	Downregulated
			HNF1α	Not affected	Downregulated
	Dong 2019 [37]	Siblinin A **	PCSK9	Downregulated	Downregulated
*HepG2*	Fan 2021 [38]	Berberine derivative (9k) **	PCSK9	Downregulated	Not reported
			LDLR	Upregulated	Not reported
	Gao 2018 [39]	Pinostrobin **	PCSK9	Downregulated	Downregulated
			LDLR	Upregulated	NSC
			SREBP2	NSC	Not reported
			HNF1α	NSC	Not reported
			FoxO3a	Upregulated	Not reported
	Fu 2020 [40]	17β-estradiol (βE2) **	PCSK9	Downregulated	Not reported
			LDLR	Upregulated	No changed
	Gu 2017 [41]	Lunasin **	PCSK9	Downregulated	Downregulated
			LDLR	Upregulated	Upregulated
			HNF1α	Not reported	Downregulated
			SREBP2	Upregulated	Upregulated
	Hwang 2020 [42]	Butein **	PCSK9	Downregulated	Downregulated
			LDLR	Upregulated	Upregulated
			HNF1α	Downregulated	Downregulated
			SREBP2	NSC	Downregulated
			HMGCR	Not reported	Downregulated
	Hwang 2021 [43]	*Capsellabursa-pastoris* *	PCSK9	Downregulated	Downregulated
			LDLR	Not affected	Downregulated
			HNF1α	Downregulated	Downregulated
			SREBP2	Downregulated	Downregulated
	Kim 2020 [44]	Piceatannol **	PCSK9	Downregulated	Downregulated
			LDLR	Upregulated	Not affected
			HNF1α	Not reported	Downregulated
			SREBP2	Not reported	Downregulated
	Lammi 2019 [45]	Lupin peptide T9 **	PCSK9	Downregulated	Not reported
			LDLR	Upregulated	Not reported
			HNF1α	Downregulated	Not reported
	Li 2020 [46]	23,24-Dihydrocucurbitacin B **	PCSK9	Downregulated	Downregulated
			LDLR	Upregulated	Upregulated
			SREBP2	Upregulated	Not reported
			HNF1α	Downregulated	Not reported
	Masagalli 2021 [47]	Moracin C **	PCSK9	Downregulated	Downregulated
	Pel 2020 [48]	5,6,7,4’-tetramethoxyflavanone **	PCSK9	Downregulated	Downregulated
			LDLR	Upregulated	NSC
			HNF1α	Not reported	Downregulated
	Pel 2017 [49]	(+)-pinoresinol **	PCSK9	Downregulated	Downregulated
	Weng 2021 [50]	Gynostemma pentaphyllum *	PCSK9	Downregulated	Downregulated
*HepG2*	Wang 2021 [51]	Gypenoside LVI **	PCSK9	Downregulated	Downregulated
			LDLR	Not reported	Not affected
			SREBP2	Not affected	Not affected
	Wu 2019 [52]	Tetrahydroprotoberberi-ne derivatives **	PCSK9	Downregulated	Downregulated
			LDLR	Upregulated	Not reported
	Wu 2021 [53]	Diallyl disulfide **	PCSK9	Downregulated	Downregulated
			LDLR	Upregulated	Upregulated
			SREBP2	Downregulated	Downregulated
			HMGCR	Downregulated	Downregulated
			HNF1α	Not affected	Not affected
	Yang 2018 [54]	Liraglutide **	PCSK9	Downregulated	Downregulated
			HNF1α	Downregulated	Downregulated
	Yang 2018 [55]	Chitosan oligosaccharides **	PCSK9	Downregulated	Downregulated
			SREBP2	Upregulated	Upregulated
			HNF1α	Upregulated	Upregulated
			FoxO3a	Upregulated	Upregulated
	Lupo 2019 [56]	Monacolin K **	PCSK9	Upregulated	Upregulated
			LDLR	Upregulated	Not reported
			HMGCR	Not reported	Upregulated
			FAS	Not reported	Upregulated
		Berberine **	PCSK9	Downregulated	Downregulated
			LDLR	Upregulated	Not reported
			HMGCR	Not reported	Downregulated
			FAS	Not reported	Downregulated
		1-deoxynojirimycin **	PCSK9	Downregulated	Downregulated
			LDLR	Upregulated	Not reported
			HMGCR	Not reported	Downregulated
			FAS	Not reported	Downregulated
	Wang 2020 [29]	Ascorbic acid **	PCSK9	Downregulated	Downregulated
			LDLR	Upregulated	Upregulated
			PPARg	Not affected	Not affected
			FoxO3a	Upregulated	Upregulated
*JLM3*	He 2017 [57]	*Actinidia chinensis* *	PCSK9	Not reported	Upregulated
			LDLR	Not reported	Upregulated

Abbreviation: HUVEC (Human Umbilical Vein Endothelial Cells); HUH7 (Human Hepatocytes); JLM3 (hepatocellular carcinoma cells); LO2 (hepatocytes); HepG2 (Human Hepatoma); NSC (not significantly changed); * Natural product; ** Plant bioactive compound. PCSK9 in Relation to FoxO3, HMGCR, PPARg, FAS, LOX-1, NOX-4, Adhesion, and Inflammatory Biomarkers.

**Table 2:** Characteristic, model validity and imprecision of selected studies on the atherogenesis biomarkers.

Biomarkers	Cell Lines	Study ID	Model Validity	Imprecision	Biomarker Validity
PCSK9	HUVEC	Wang 2019 [27]	Low	Low	Unclear
	Huh7	Mbikay 2014 [28]	Low	Low	Low
		Wang 2020 [29]	Low	Low	Low
	LO2	Jing 2019 [30]	Low	Low	Unclear
	JLM3	He 2017 [57]	Unclear	Low	Low
	HepG2	Aggrey 2019 [31]	Unclear	Unclear	Unclear
		Ahn 2019 [32]	Unclear	Low	Unclear
		Cameron 2008 [33]	Low	Low	Low
		Chae 2018 [34]	Unclear	Low	Low
		Chen 2016 [35]	Low	Low	Unclear
		Choi et 2017 [36]	Low	Unclear	Low
		Dong 2019 [37]	Unclear	Low	Unclear
		Fan 2021 [38]	Low	Low	Unclear
		Gao 2018 [39]	Low	Low	Low
		Fu 2020 [40]	Unclear	Low	Unclear
		Gu 2017 [41]	Low	Low	Unclear
		Hwang 2020 [42]	Low	Low	Unclear
		Hwang 2021 [43]	Low	Unclear	Low
		Kim 2020 [44]	Low	Low	Low
		Lammi 2019 [45]	Low	Low	Unclear
		Li 2020 [46]	Low	Low	Unclear
		Masagalli 2021 [47]	Low	Low	Unclear
		Pel 2020 [48]	Unclear	Unclear	Unclear
		Pel 2017 [49]	High	Unclear	Low
		Weng 2021 [50]	Low	Unclear	Low
		Wang 2020 [51]	Low	Low	Unclear
		Wu 2019 [52]	Unclear	Low	Low
		Wu 2021 [53]	Low	Low	Unclear
		Yang 2018 [54]	Low	Low	Unclear
		Yang 2018 [55]	Low	Low	Unclear
		Lupo 2019 [56]	Unclear	Low	Low
		Wang 2020 [29]	Low	Low	Low
LDLR	HUVEC	Wang 2019 [27]	Low	Low	High
	Huh7	Mbikay 2014 [28]	Low	Low	Low
		Wang 2020 [29]	Low	Low	Low
	LO2	Jing 2019 [30]	Low	Unclear	Unclear
	JLM3	He 2017 [57]	Unclear	Low	Low
	HepG2	Aggrey 2019 [31]	Unclear	Unclear	Unclear
		Cameron 2008 [33]	Low	Low	Low
		Chae 2018 [34]	Unclear	Low	Low
		Chen 2016 [35]	Low	Low	Unclear
		Choi 2017 [36]	Low	Unclear	Unclear
		Fan 2021 [38]	Low	Low	Unclear
		Gao 2018 [39]	Low	Low	Low
		Fu 2020 [40]	Unclear	Low	Unclear
		Gu 2017 [41]	Low	Low	Unclear
		Hwang 2020 [42]	Low	Low	Unclear
		Hwang 2021 [43]	Low	Unclear	Low
		Kim 2020 [44]	Low	Low	Low
		Lammi 2019 [45]	Low	Low	Unclear
		Li 2020 [46]	Low	Low	Low
		Pel 2017 [49]	High	Unclear	Unclear
		Wang 2021 [51]	Low	Low	Unclear
		Wu 2019 [52]	Unclear	Low	Low
		Wu 2021 [53]	Low	Low	Unclear
		Lupo 2019 [56]	Unclear	Low	Low
		Wang 2020 [29]	Low	Low	Low
SREBP	HUVEC	Wang 2019 [27]	Low	Low	Unclear
	Huh7	Mbikay 2014 [28]	Low	Low	Unclear
	LO2	Jing 2019 [30]	Low	Unclear	Unclear
	HepG2	Choi et 2017 [36]	Low	Unclear	Unclear
		Gao 2018 [39]	Low	Low	Unclear
		Gu 2017 [41]	Low	Low	Unclear
		Hwang 2020 [42]	Low	Low	Unclear
		Hwang 2021 [43]	Low	Unclear	Low
		Kim 2020 [44]	Low	Low	Unclear
		Li 2020 [46]	Low	Low	Unclear
		Wang 2021 [51]	Low	Low	Unclear
		Wu 2021 [53]	Low	Low	Unclear
		Yang 2018 [55]	Low	Low	Unclear
HNF1α	HepG2	Choi 2017 [36]	Low	Unclear	Unclear
		Gao 2018 [39]	Low	Low	Unclear
		Gu 2017 [41]	Low	Low	Unclear
		Hwang 2020 [42]	Low	Low	Unclear
		Hwang 2021 [43]	Low	Unclear	Low
		Kim 2020 [44]	Low	Low	Unclear
		Li 2020 [46]	Low	Low	Low
		Pel 2020 [48]	Unclear	Unclear	Unclear
		Wu 2021 [53]	Low	Low	Unclear
		Yang 2018 [55]	Low	Low	Unclear
FoxO3a	Huh7	Wang 2020 [29]	Low	Low	Low
	HepG2	Gao 2018 [39]	Low	Low	Unclear
		Yang 2018 [55]	Low	Low	Unclear
		Wang 2020 [29]	Low	Low	Low
HMGCR	HepG2	Hwang 2020 [42]	Low	Low	Unclear
		Wu 2021 [53]	Low	Low	Unclear
		Lupo 2019 [56]	Unclear	Low	Low
PPARg	Huh7	Wang 2020 [29]	Low	Low	Unclear
	HepG2	Wang 2020 [29]	Low	Low	Unclear
FAS	HepG2	Lupo 2019 [56]	Unclear	Low	Unclear
LOX-1	HUVEC	Wang 2019 [27]	Low	Low	Unclear
NOX-4	HUVEC	Wang 2019 [27]	Low	Low	Unclear
ICAM	HUVEC	Wang 2019 [27]	Low	Low	Unclear
VCAM	HUVEC	Wang 2019 [27]	Low	Low	Unclear
(IL)-1α,	HUVEC	Wang 2019 [27]	Low	Low	Unclear
IL-1β	HUVEC	Wang 2019 [27]	Low	Low	Unclear
IL-6	HUVEC	Wang 2019 [27]	Low	Low	Unclear
MCP-1	HUVEC	Wang 2019 [27]	Low	Low	Unclear
CXCL-1	HUVEC	Wang 2019 [27]	Low	Low	Unclear
CXCL-2	HUVEC	Wang 2019 [27]	Low	Low	Unclear

Imprecision interpretation: Low = no concern, Unclear = not enough information to make judgement, High risk = there is a concern of high risk. Model validity interpretation: Low = all domains clearly reported, and there were no additional concerns. Unclear = Any domain was unclear, but not high risk. High risk = there is a concern of high risk.

**Figure A1:**
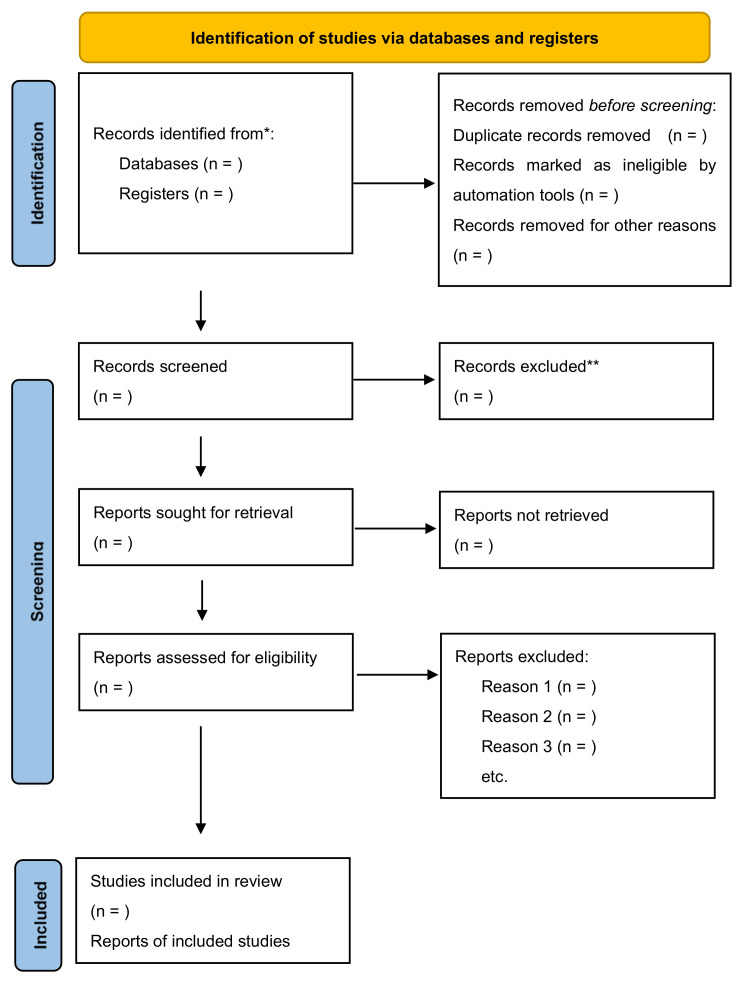
PRISMA 2020 Checklist. * Consider, if feasible to do so, reporting the number of records identified from each database or register searched (rather than the total number across all databases/registers). ** If automation tools were used, indicate how many records were excluded by a human and how many were excluded by automation tools.

## Data Availability

Data is available in a publicly accessible repository.

## Appendix B

**Table A1:** Search Strategies.

Search	Query
**PubMed (*k* = 491)**	
#4	**(#1 AND #2 AND #3 AND #4)**
#3	Cell OR Cells OR Endothelial cell OR Endothelial cells [tiab]
#2	PCSK9 Inhibition [tiab]
#1	Proprotein Convertase Subtilisin Kexin 9 Inhibitor*[tiab] OR PCSK9 Inhibitor*[tiab]
**Science Direct (*k* = 3259)**	
#3	**(#1 AND #2 AND #3)**
#2	Topic: Cell* OR Endothelial Cell*
#1	Topic: PCSK9 Inhibitor* OR PCSK9 Inhibition*
**Scopus (*k =* 4653)**	
**#3**	**(#1 AND #2 AND #3 AND #4)**
#2	Topic: PCSK9 Inhibitor* AND (Endothelial Cell*)
#1	Topic: (PCSK9 Inhibitor* AND Cell*

## Appendix C

**Table A2:** Imprecision tool.

	Signalling Question	Notes	Answer
**TECHNICAL REPORTING**	**1. How many technical repeats were performed per experiment?**	Intra-assay variability	Free text
	**2. Observer variability: Did the experiment give the same result when repeated?**	Inter-assay variability	Free text
	**3. Is it clear whether the technical repeat is true or a combination of technical and observer variability?**		Yes/no/not applicable/unclear or not reported
	**4. Did the result include a measure of variability? Or was the data presented as a scatter plot?**	EB = error bars (unclear error), SE = standard error, SEM = standard error of the mean, SD = standard deviation CI = confidence intervals	Free text
	**5. Did the authors pool data between experiments? If so, was heterogeneity measured to test that pooling was appropriate?**	(Important when using multiple patient/animal samples)	Yes/no/not applicable/unclear or not reported
	**Overall reporting rating**	Low = no concern for bias. Unclear = insufficient data to make a judgment. High risk = there is a concern of high risk. If 1,2 and 4 are fulfilled this can be given a low rating for this review.	**Low/Unclear/High**
**SAMPLE SIZE**	**6. Were sample sizes calculated?**	For the given experiment/effect did the authors calculate the number of repeats that would be required for significance?	Yes/no/not applicable/unclear or not reported
	**7. How were indeterminate results, missing results, and outliers handled?**		Free text
	**8. Did the study have sufficient statistical power?**	Yes: clearly meets the sample size. Likely: >10 repeats with inter-assay repeats. Unclear: >10 repeats, no inter-assay repeats. No: ≤10 technical repeats.	Yes/no/unclear/likely
	**Did the study have sufficient statistical power? Justification**	Based on questions 6-8	Free text
	**Overall sample size rating**	Low = no concern (or likely statistical power or Unclear statistical power plus variability reported). Unclear: not enough information to make judgement and no high risk for 6-8. High risk: there is a concern of high risk for 6-8.	**Low/Unclear/High**
**STATISTICAL TEST**	**9. Description of statistical methods and assumptions.**	P S TT = Paired student *t*-test; US TT = unpaired student t test; x2-test = XT; Fishers exact test = FET; others possible	Free text
	**10. Were the statistical tests appropriate?**	In this review *t*-tests were the predominant test (other statistical tests are possible). A paired tt-testis the most appropriate test for comparisons between the same cell lines or non-human models, because these are assumed to be homogeneous populations. An unpaired t test should be used for comparisons between primary cultures, human tissues, or different mutants or strains, because these will be heterogeneous populations.	Yes/no/not applicable/unclear or not reported
	**Were the statistical tests appropriate? Justification**		Free text
	**11. Evidence of data dredging**	https://en.wikipedia.org/wiki/Data_dredging (accessed on 31 August 2022).	Yes/no/not applicable/unclear or not reported
	**Statistical test rating**	**Low = no concern. Unclear = not enough information to make judgement. High risk = there is a concern of high risk**	**Low/Unclear/High**
	**Other Concerns**		Free text

**Table A3:** Imprecision Tool Assessments on Selected Studies.

First Author Surname and Year	Experiment	1.	2.	3.	4.	5.	Reporting Rating	6.	7.	8.	Sample Size Rating	9.	10.	11.	Statistical Test Rating	Overall Rating	Justification
***HUVECs (Human Umbilical Vein Endothelial Cells)***																	
Wang et al. (2019)	Ginkgolide B	3	NR	NR	SEM	NA	*Low*	NA	NR	NO	*UNR*	S TT	YES	UNR	*Low*	**Low**	The observer variability was not reported. The sample size rating is unclear. It is not applicable because it is not a common practice to calculate sample size in cell culture studies.
***Huh7 (Human Hepatocytes)***																	
Wang et al. (2020)	Ascorbic acid	5	NR	NR	SE	NA	*Low*	NA	NR	NO	*UNR*	P TT	YES	UNR	*Low*	**Low**	The technical repeats were high. The observer variability was not reported. Sample size rating is unclear. It is not applicable because it is not a common practice to calculate sample size in cell culture studies.
Mbikay et al. (2014)	Quercetin-3-glucoside	3	NR	NR	SEM	NA	*Low*	NA	NR	NO	*UNR*	S TT	YES	UNR	*Low*	**Low**	The observer variability was not reported. Sample size rating is unclear. It is not applicable because it is not a common practice to calculate sample size in cell culture studies.
***LO2 (hepatocytes)***																	
Jing et al. (2019)	Resveratrol	3	NR	NR	SEM	NA	*Low*	NA	NR	NO	*UNR*	ANOVA	YES	UNR	*Low*	**Low**	The observer variability was not reported. The sample size rating is unclear. It is not applicable because it is not a common practice to calculate sample size in cell culture studies.
***HepG2 (Human Hepatoma)***																	
Fan et al. (2021)	Berberine derivative (9k)	3	NR	NR	SEM	NA	*Low*	NA	NR	NO	*UNR*	S TT	YES	UNR	*Low*	**Low**	The observer variability was not reported. One domain was reported unclear. Sample size rating is unclear. It is not applicable because it is not a common practice to calculate sample size in cell culture studies. The observer variability was not reported. One domain was reported unclear. Sample size rating is unclear. It is not applicable because it is not a common practice to calculate sample size in cell culture studies.
Masagalli et al. (2021)	Moracin C and Its Derivatives with a 2-arylbenzofuran Motif (Compound 7)	3	NR	NR	SEM	NA	*Low*	NA	NR	NO	*UNR*	Dunnet TT	YES	UNR	*Low*	**Low**	
Wang et al. (2020)	Gypenoside LVI	3	NR	NR	SD	NA	*Low*	NA	NR	NO	*UNR*	S TT	YES	UNR	*Low*	**Low**	
Fu et al. (2020)	17β-estradiol (βE2)	3	NR	NR	SEM	NA	*Low*	NA	NR	NO	*UNR*	Duncan T	YES	UNR	*Low*	**Low**	
Hwang et al. (2020)	Butein	3	NR	NR	SD	NA	*Low*	NA	NR	NO	*UNR*	S TT	YES	UNR	*Low*	**Low**	
Kim et al. (2020)	Piceatannol	3	NR	NR	SD	NA	*Low*	NA	NR	NO	*UNR*	S TT	YES	UNR	*Low*	**Low**	
Li et al. (2020)	23,24- Dihydrocucurbitacin B	3	NR	NR	SD	NA	*Low*	NA	NR	NO	*UNR*	S TT	YES	UNR	*Low*	**Low**	
Ahn et al. (2019)	Sophora tonkinensis (erybraedin D-compound 16)	3	NR	NR	SEM	NA	*Low*	NA	NR	NO	*UNR*	D TT	YES	UNR	*Low*	**Low**	
Dong et al. (2019)	Siblinin A	3	NR	NR	SD	NA	*Low*	NA	NR	NO	*UNR*	Post Hoc T	YES	UNR	*Low*	**Low**	
Lammi et al. (2019)	Lupin peptide T9 (GQEQSHQDEGVIVR)	4	NR	NR	SD	NA	*Low*	NA	NR	NO	*UNR*	Dunnet T	YES	UNR	*Low*	**Low**	
Lupo et al. (2019)	red yeast rice RYR (monacolin K),	3	NR	NR	SD	NA	*Low*	NA	NR	NO	*UNR*	S TT	YES	UNR	*Low*	**Low**	
	Berberis aristata cortex BCE (Berberine)	3	NR	NR	SD	NA	*Low*	NA	NR	NO	*UNR*	S TT	YES	UNR	*Low*	**Low**	
	Morus alba leaves extract MLE (1-deoxynojirimycin)	3	NR	NR	SD	NA	*Low*	NA	NR	NO	*UNR*	S TT	YES	UNR	*Low*	**Low**	
Wu et al. (2019)	tetrahydroprotoberberine derivatives (THPBs) (Compound 22)	3	NR	NR	SEM	NA	*Low*	NA	NR	NO	*UNR*	S TT	YES	UNR	*Low*	**Low**	The observer variability was not reported. One domain was reported unclear. The sample size rating is unclear. It is not applicable because it is not a common practice to calculate sample size in cell culture studies.
Chae et al. (2018)	Saucinone	3	NR	NR	SEM	NA	*Low*	NA	NR	NO	*UNR*	D TT	YES	UNR	*Low*	**Low**	
Yang et al. (2018)	Liraglutide	3	NR	NR	SE	NA	*Low*	NA	NR	NO	*UNR*	S TT	YES	UNR	*Low*	**Low**	
Yang et al. (2018)	Chitosan oligosaccharides	3	NR	NR	SD	NA	*Low*	NA	NR	NO	*UNR*	S TT	YES	UNR	*Low*	**Low**	
Gu et al. (2017)	Lunasin	3	NR	NR	SEM	NA	*Low*	NA	NR	NO	*UNR*	ANOVA	YES	UNR	*Low*	**Low**	
Chen et al. (2016)	Salvia miltiorrhiza Bunge (Tanshinone IIA)	3	NR	NR	SD	NA	*Low*	NA	NR	NO	*UNR*	D TT	YES	UNR	*Low*	**Low**	
Cameron et al. (2008)	Berberine	3	NR	NR	SEM	NA	*Low*	NA	NR	NO	*UNR*	P TT	YES	UNR	*Low*	**Low**	
Gao et al. (2018)	Pinostrobin	3	YES	NR	U	NA	*Low*	NA	NR	NO	*UNR*	Post Hoc T (Dunnet)	YES	UNR	*Low*	**Low**	The only article that reported on observer variability was reported. The measurement of variability is not clear. One domain was reported unclear. The sample size rating is unclear. It is not applicable because it is not a common practice to calculate sample size in cell culture studies.
Hwang et al. (2021)	Capsella Bursa-Pastoris	NR	NR	NR	SD	NA	*UNR*	NA	NR	NO	*UNR*	S TT	YES	UNR	*Low*	**UNR**	Two domains were reported as unclear. The number of technical repeats was not mentioned in the article. The observer variability was not reported. Sample size rating is unclear. It is not applicable because it is not a common practice to calculate sample size in cell culture studies.
Weng et al. (2021)	Gynostemma pentaphyllum [dammarane-type glycosides (2, 3, 15)]	NR	NR	NR	SD	NA	*UNR*	NA	NR	NO	*UNR*	ANOVA	YES	UNR	*Low*	**UNR**	
Pel et al. (2020)	Chromolaena odorata – involve many extraction & many compounds (Compound 6)	NR	NR	NR	SEM	NA	*UNR*	NA	NR	NO	*UNR*	Dunnet TT	YES	UNR	*Low*	**UNR**	
Choi et al. (2017)	Welsh onion (Allium fistulosum L. [family Amaryllidaceae])	NR	NR	NR	SD	NA	*UNR*	NA	NR	NO	*UNR*	S TT	YES	UNR	*Low*	**UNR**	
Choi et al. (2017)	Welsh onion (Allium fistulosum L. [family Amaryllidaceae])	NR	NR	NR	SD	NA	*UNR*	NA	NR	NO	*UNR*	S TT	YES	UNR	*Low*	**UNR**	
Pel et al. (2017)	Schisandra chinensis (Turcz.) (Compound 10)	NR	NR	NR	SEM	NA	*UNR*	NA	NR	NO	*UNR*	Dunnet TT	YES	UNR	*Low*	**UNR**	
Aggrey et al. (2019)	Nauclea latifolia (Compound 5)	3	NR	NR	SEM	NA	*Low*	NA	NR	NO	*UNR*	NR	NO	UNR	*UNR*	**UNR**	The observer variability was not reported. Two domains were reported unclear. The sample size rating is unclear. It is not applicable because it is not a common practice to calculate sample size in cell culture studies. The statistical test rating was reported unclear because no analysis was reported on the comparison.
***JLM3 (hepatocellular carcinoma cells)***																	
He et al. (2017)	Actinidia chinensis Planch root extract	3	NR	NR	SD	NA	*Low*	NA	NR	NO	*UNR*	S TT	YES	UNR	*Low*	**Low**	The observer variability was not reported. The sample size rating is unclear. It is not applicable because it is not a common practice to calculate sample size in cell culture studies.

UNR= unclear or not reported; NR = not reported; U = unclear; L = likely; PS TT = Paired student *t*-test; US TT = unpaired student t test; x2-test =XT; Fishers exact test = FET; TT = t test or student’s t test.

## Appendix D

**Table A4:** Model validity tool.

Signalling Question	Notes	Answer
**1. Ethical statement**	Was an ethical statement provided for animal handling?	Yes/NR—add details to justification
**2. Clear description of model details**	Brief description of basic model followed by source, species, strain sex, developmental stage, age, passage number, etc.).	Free text
**3. Is the model transgenic?**	Whether purchased or created.	Yes/no/unclear
**4. Clear description of the routine maintenance of the model**		Free text
**5. Further preparation of the model for experimentation**	Description of how model was manipulated to obtain result: to include preparation for imaging, how daughter or mother organelle were induced to differentiate. This should be used to make it clear how result was derived.	Free text
**6. If the model is of an adult stem cell do the authors prove this?**	Cells must be capable of dividing and renewing for long periods; undifferentiated; multipotent.	NA/partial/NR/yes/no—add details to justification.
**7. Cell lines: were they routinely checked for the absence of mycoplasma or contaminants?**		Yes/no/NR
**8. Primary cultures: was the tissue of origin tracked/proven?**		Yes/no/NR
**9. Additional comments/concerns**		NA/partial/No/yes—add details to justification.
**Overall rating/reporting of model.**	**Low = all domains clearly reported, and there were no additional concerns. Unclear = Any domain was unclear, but not high risk. High risk = there is a concern of high risk. Note that for this review routine maintenance was not essential for low order organisms.**	**High/Low/Unclear or not reported**
**Justification**	**Text to justify why model was given unclear or high rating. Additional text for details regarding questions 1, 6-9.**	**Free text.**

**Table A5:** Model Validity Tool Assessments of Selected Studies.

Study ID	1.	2.	3.	4.	5.	6.	7.	8.	9.	Overall Rating/ Reporting of Model	Justification
***HUVECs (Human Umbilical Vein Endothelial Cells)***											
Wang et al. (2019)	NR	Human Umbilical Vein Endothelial Cells (from American Type Culture Collection; ATCC)	YES	Cells were cultured under standard culture conditions in DMEM containing 10% heat Inactivated FBS, 2 Mm glutamine, and antibiotics (100 U/mL).	To study the impact of oxidatively modified-LDL on various biochemical and molecular parameters, HUVECs were incubated with Ox-LDL (25–100 µg/mL).	NA	NR	NR	NA	**Low**	No description of its routine maintenance nor check for contaminants
***Huh7 (Human Hepatocytes)***											
Wang et al. (2020)	NR	Huh7 was obtained from ATCC.	YES	Huh7 cells, the human hepatic cell lines, were cultured in high glucose DMEM containing 10% FBS, 50 mg/mL penicillin and streptomycin, and 2 Mm glutamine.	After reaching, 80% confluence, cells received treatment in medium containing 2% FBS.	NA	NR	NR	NA	**Low**	No clear description of model nor its routine maintenance. No inducer was given to stimulate the cells.
Mbikay et al. (2014)	NR	Huh7 human liver cells were obtained from the Japanese Collection of Research Bioresources	YES	Huh7 cell incubations were carried out at 37 °C in a humidified 5% CO_2_–95% air atmosphere in DMEM containing 10% FBS for maintenance or LPDS for experiments, and 50 g/mL gentamycin	LPDS was used for experiments, and 50 µg/mL gentamycin; they were incubated overnight and then treated or not with Q3G, or simvastatin, or both, at defined concentrations, and for defined lengths of time.	NA	NR	NR	NR	**Low**	
***LO2 (hepatocytes)***											
Jing et al. (2019)	NR	Human L02 hepatocytes were obtained from the Cell Bank of the Chinese Academy of Sciences (Shanghai, China)	YES	LO2 hepatocytes were cultured overnight in DMEM, supplemented with 10% FBS 100 U/mL of penicillin, and 100 μg/mL of streptomycin at 37 °C in a humidified atmosphere of 5% CO_2_ and 95% air	To induce cellular steatosis, the cells were exposed to a mixture of FFA (oleate: palmitate = 2:1) at a final concentration of 1 mM for 24 h	NA	NR	NR	NR	**Low**	No description of its routine maintenance nor check for contaminants
***HepG2 (Human Hepatoma)***											
Fan et al. (2021)	NR	NR	NO	Cells were cultured in Eagle EMEM, supplemented with 10% FBS 1% nonessential amino acids, and 1% sodium pyruvate	The stable pGL4-PCSK9-P transfected HepG2 cells, named as PCSK9p-Luc HepG2 cells and used as PCSK9 transcriptional inhibitor HTS assay, were cultured in MEM supplemented with 10% FBS, 1% nonessential amino acids, 1% sodium pyruvate and additional G418 (700 mg/mL, Invitrogen). Cells were maintained at 37 °C in the presence of 5% CO_2_.	NA	NR	NR	NR	**Low**	No description of the origin or source of cell lines.
Masagalli et al. (2021)	NR	HepG2 cells were obtained from the Chinese Academy of Cell Resource Center (Xiangf bio, Shanghai, China)	YES	Cells were maintained in low glucose DMEM containing 10% FBS at 37 °C under 5% CO_2_ atmosphere	During experiment, cells were seeded in corresponding culture vessels, after reaching 50- 60% confluence, culture media were changed to DMEM supplemented with 5% LPDS while the control group changed to fresh 5% FBS.	NA	NR	NR	NR	**Low**	No description of its routine maintenance nor check for contaminants
Wang et al. (2021)	NR	HepG2 cells were obtained from the Chinese Academy of Cell Resource Center (Xiangf bio, Shanghai, China)	YES	Cells were maintained in low glucose DMEM containing 10% FBS at 37 °C under 5% CO_2_ atmosphere	During the experiment, cells were seeded in corresponding culture vessels, after reaching 50–60% confluence, culture media were changed to DMEM supplemented with 5% LPDS, while the control group changed to fresh 5% FBS.	NA	NR	NR	NR	**Low**	
Fu et al. (2020)	NR	HepG2 cells (ATCC, USA)	YES	Cells were maintained at 37 °C in phenol red-free DMEM supplemented with 10% FBS, 100 IU/mL penicillin, and 100 µg/mL streptomycin	For all assays, the cells were pre-treated with 1µM G15 for 15 min prior to the addition of βE2 to block GPER action. After a series of wash steps with PBS, internalized AF−PCSK9 was directly observed under an inverted fluorescence microscope, and the fluorescence intensity of AF−PCSK9 in isopropyl alcohol was detected by a SpectraMax M5 reader and reported in RFUs.	NA	NR	NR	NR	**UNR**	
Hwang et al. (2020)	NR	HepG2 cells (HB-8065) were purchased from ATCC (Manassas, VA, USA).	YES	The cells were cultured with DMEM high glucose; supplemented with 10% FBS and 1% antibiotic and antimycotic solution in an incubator (37 °C and 5% CO_2_)	After 24 h, the media were changed to either DMEM supplemented with FBS or delipidated serum (DLPS)17 (day 1). The media were then changed to media supplemented with either FBS or DLPS + butein	NA	NR	NR	NR	**Low**	No description of the model nor its routine maintenance
Kim et al. (2020)	NR	HepG2 cells (HB-8065) were purchased from the American Type Culture Collection (Manassas, VA, USA).	YES	The cells were cultured with high glucose DMEM supplemented with 10% FBS and 1% antibiotics in a humidified atmosphere of 5% CO_2_ at 37 °C	After reaching ≈50% confluence (day 0), the medium was changed to either DMEM supplemented with FBS or DLPS, andthe next day, the medium was changed to either FBS or DLPS supplemented DMEM with piceatannol alone or in combination with rosuvastatin or simvastatin	NA	NR	NR	NR	**Low**	
Li et al. (2020)	NR	HepG2 cells (catalogue number: HB-8065, ATCC, Manassas, VA, USA)	YES	Cells were maintained in DMEM with 10% FBS and incubated under a humidified atmosphere of 95% O2 and 5% CO_2_ at 37 °C. The cells were subcultured once every 2 days.	LDL and LPDS were separated from the pooled plasma of healthy volunteers by ultracentrifugation and were then dialyzed in dialysis buffer and phosphate-buffered saline (PBS). After specific treatments, the culture medium was changed to DiI-LDL DMEM (20 μg/mL) or changed to 2% LPDS.	NA	NR	NR	NR	**Low**	Human plasma was obtained from Shanghai Xuhui Central Hospital, China, after informed consent was obtained and approval was granted by the Ethics Committee. The procedures conformed to the principles outlined in the Declaration of Helsinki Cells within 4–11 passages were used for experiments.
Ahn et al. (2019)	NR	HepG2 cell line was obtained from the Korea Research Institute of Bioscience and Biotechnology (South Korea)	YES	Cells were grown in EMEM, supplemented with 10% FBS and 100 U/mL penicillin/streptomycin sulfate. Cells were incubated in a humidified incubator at 37 °C in a 5% CO_2_ atmosphere	NR	NA	NR	NR	NR	**UNR**	No description of the model nor its routine maintenance
Dong et al. (2019)	NR	HepG2 cells were obtained from the ATCC	YES	Cells were cultured in DMEM supplemented with 10% FBS and 1% penicillin/streptomycin solution. All cells were incubated in a cell culture chamber at 37 °C under a humidified atmosphere with 5% CO_2_.	NR	NA	NR	NR	NR	**UNR**	
Lammi et al. (2019)	NR	The HepG2 cell line was bought from ATCC (HB-8065, ATCC from LGC Standards, Milan, Italy)	YES	The HepG2 cell line was cultured in DMEM high-glucose with stable L-glutamine supplemented with 10% FBS, 100 U/mL penicillin, and 100 μg/mL streptomycin (complete growth medium) and incubated at 37 °C under 5% CO_2_ atmosphere	Cells at a 70–90% confluence were transfected with the mixture containing 1.0 µg pcDNA3+PCSK9D374Y-FLAG plasmid and 2.0 µL TurboFect Transfection Reagent in 100 µL of serum-free DMEM for 48 h. After 24 h, transfected HepG2 cells were treated with peptide T9 (100 µM) and incubated for 24 h at 37 °C under 5% CO_2_ atmosphere	NA	NR	NR	NR	**Low**	HepG2 cells were used for no more than 20 passages after thawing
Lupo et al. (2019)	NR	NR	NO	HepG2 was cultured in MEM supplemented with 10% FCS, L-glutamine, sodium-pyruvate and non-essential amino acids, penicillin/streptomycin at 37 °C in a humidified atmosphere of 5% CO_2_ and 95% air.	NR	NA	NR	NR	NR	**UNR**	No description of the model nor its routine maintenance
Wu et al. (2019)	NR	NR	NO	The cell line HepG2 was maintained in DMEM, supplemented with 10% FBS, 100 units/mL penicillin, and 100 mg/mL streptomycin and cultured in a 37 °C _2_incubator with 5% CO_2_ in the air	NR	NA	NR	NR	NR	**UNR**	
Chae et al. (2018)	NR	HepG2 human hepatocellular liver cell line was obtained from the Korea Research Institute of Bioscience and Biotechnology (South Korea)	YES	Cells were grown in EMEM) containing 10% FBS and 100U/Mlpenicillin/streptomycin sulfate. Cells were incubated in a humidified 5% CO_2_ atmosphere at 37 °C.	NR	NA	NR	NR	NR	**UNR**	No description of the model nor its routine maintenance
Yang et al. (2018)	NR	The human hepatoma cell line, HepG2, was obtained from Cell Resource Center, IBMS, CAMS/PUMC (Beijing, China)	YES	Cells were cultured in DMEM containing 10% FBS 1% non-essential amino acids (NEAA) and 1% penicillin–streptomycin at 37 °C, 5% (*v*/*v*) CO_2_.	HepG2 cells were serum-starved for 18 h and then treated with liraglutide at various concentrations for 24 h	NA	NR	NR	NR	**Low**	No description of its routine maintenance nor check for contaminants
Yang et al. (2018)	NR	The HepG2 cell line was obtained from the American Type Culture Collection (ATCC; Manassas, VA	YES	The cells were cultured in DMEM containing 10% FBS at 37 °C and 5% CO_2_ atmosphere. After reaching 70–80% confluence, the HepG2 cells were pre-treated with vehicle or COS (50–200 μg/mL) in DMEM with 4% FBS for 24 h.	After reaching 70–80% confluence, the HepG2 cells were pre-treated with vehicle or COS (50–200 μg/mL) in DMEM with 4% FBS for 24 h	NA	NR	NR	NR	**Low**	
Gu et al. (2017)	NR	Human hepatic HepG2 cells were obtained from China Infrastructure of Cell Line Resources (Beijing, China)	YES	Cells were cultured in a complete medium consisting of MEM supplemented with penicillin (100 U/mL), streptomycin (100 μg/mL) and 10% FBS in a humidified 5% CO_2_ atmosphere at 37 °C.	OptiMEM media was used in the Lunasin dose-response and time-course experiments to measure the amount of PCSK9 secreted into the culture media and LDLR expression	NA	NR	NR	NR	**Low**	
Chen et al. (2016)	NR	HepG2 cells were obtained from the Bioresource Collection and Research Center (Hsinchu, Taiwan)	YES	Cells were maintained in a DMEM medium containing 10% FBS.	The cells were seeded and cultured in normal serum medium overnight; then, the medium was changed to DMEM supplemented with 5% LPDS and was cultured for 24 h.	NA	NR	NR	NR	**Low**	
Cameron et al. (2008)	NR	HepG2 cells (European collection of cell cultures, Wiltshire, UK)	YES	Cells were maintained in MEM, containing penicillin (50 U/mL), streptomycin (50 (g/mL), l-glutamine (2 mM) and 10% fetal calf serum (FCS) in a humidified atmosphere (37 °C, 5% CO_2_)	OptiMEM (Gibco) media was used instead of media containing 10% LPDS in the dose-response and time-course experiments	NA	NR	NR	NR	**Low**	No description of its routine maintenance nor check for contaminants
Gao et al. (2018)	NR	The HepG2 cell line was obtained from the Bioresource Collection and Research Center 124 (Hsinchu, Taiwan).	YES	The cells were cultured in DMEM containing 10% FBS and 1x non-essential amino acid (NEAA) solution.	For compound treatment, the cells were seeded in a culture medium for 24 h. The medium was replaced with 127 DMEM supplemented with 5% LPDS) for 24 h incubation	NA	NR	NR	NR	**Low**	
Hwang et al. (2021)	NR	HepG2 cells (HB-8065; ATCC, Manassas, VA, USA)	YES	Cells were cultured in high-glucose DMEM supplemented with 10% FBS and 1% antibiotic and anti-mycotic solution DLPS was prepared.	After reaching 70–80% confluence, the cells were seeded in well plates (day 0), and the medium was changed to either DMEM supplemented with FBS or DMEM supplemented with DLPS (day 1). After 24 h incubation, the medium was changed to media supplemented with either FBS or DLPS, and simultaneously treated with either samples (CBE or chemical compounds) or DMSO (day 2). After an additional hour of incubation (day 3), the cells were either washed with cold DPBS or collected for subsequent experiments.	NA	NR	NR	NR	**Low**	
Weng et al. (2021)	NR	Human hepatoma HepG2 cells were purchased from the Chinese Academy of Sciences (Shanghai, China).	YES	HepG2 cells were cultured in DMEM (low glucose), and media were supplemented with 10% FBS and 1% penicillin-streptomycin at 37 °C in a humid atmosphere with 5% CO_2_.	The cells were inoculated in 12-well plates at 1 × 105 /well, which cultured in DMEM (low glucose) containing 10% FBS at 37 °C with 5% CO_2_. After cell adherence, the media were replaced with DMEM containing 5% LPDS and incubated for 23 h in the incubator	NA	NR	NR	NR	**Low**	
Pel et al. (2020)	NR	The HepG2 human hepatocellular liver cell line was provided by the Korea Research Institute of Bioscience and Biotechnology, Republic of Korea	YES	Cells were grown in EMEM containing 10% FBS and 100 U/Ml penicillin/streptomycin sulfate. Cells were incubated in a humidified 5% CO_2_ atmosphere at 37 °C.	NR	NA	NR	NR	NR	**UNR**	No description of the model nor its routine maintenance
Choi et al. (2017)	NR	HepG2 cells (HB-8065; ATCC, Manassas, VA, USA)	YES	Cells were cultured with high glucose DMEM supplemented with 10% FBS and 1% antibiotic and antimycotic solution	After reaching 70–80% confluence, the cells were seeded in 96-well plates (day 0), and the medium was changed to either DMEM supplemented with FBS or DMEM supplemented DLPS; day 1. DLPS was prepared as previously described.26 After 24 hours of incubation, the medium was changed to media supplemented with either FBS or DLPS.	NA	NR	NR	NR	**Low**	No description of its routine maintenance nor check for contaminants
Pel et al. (2017)	NR	NR	NO	NR	NR	NA	NR	NR	NR	**High**	All domains were not reported/not applicable.
Aggrey et al. (2019)	NR	HepG2 cells (ATCC HB-8065)	YES	Cells were maintained in DMEM supplemented with 10% FBS. Cells were incubated under a humidified atmosphere of 95% O2 and 5% CO_2_ at 37 °C	NR	NA	NR	NR	NR	**UNR**	No description of the model nor its routine maintenance
***JLM3 (hepatocellular carcinoma cells)***											
He et al. (2017)	NR	RAW264.7 murine macrophages were obtained from the Korean Research Institute of Bioscience and Biotechnology (Daejeon, Korea)	YES	Cells were grown in RPMI 1640 medium supplemented with 10% FBS and 100 U/ML penicillin/streptomycin sulfate.	NR	NA	NR	NR	NR	**UNR**	No description of the model nor its routine maintenance

UNR= unclear or not reported; NR = not reported; U = unclear; NA = not applicable; DMEM= Dulbecco’s modified Eagle’s medium; FBS= Fetal Bovine Serum; LPDS= lipoprotein-deficient serum; EMEM= eagle’s minimal essential medium.

## Appendix E

**Table A6:** Marker validity tool.

Domain	Signalling Question	Notes	Answer
**Validation of Marker**	**1. Functional validation according to report aims or methods.**		Free text
	**2. Cellular localisation according to Genecard confidence 5 or cellular components according to Flybase**	http://www.genecards.org/ (accessed on 31 August 2022). http://flybase.org/ (accessed on 31 August 2022).	Free text or NA/NR
	**3. gene ontology—cellular component terms according to Genecards**	http://www.genecards.org/ (accessed on 31 August 2022).	Free text or NA/NR
	**4. Do the authors present data for functional validation in results?**	This includes: Is the marker in the correct location? Any functional experiments?	Yes/NR/Referenced If yes add free text to justification.
	**5. Were co-localisation experiments performed with a second marker/was the result confirmed with a second marker?**		
	**Validation rating**	Low= no concerns. Unclear/not reported = insufficient data to make a judgement or not reported High risk = there are concerns	**Low/UNR/High/** **Referenced** **If UNR/High add free text to justification**
**Controls**	**6. Is there an appropriate positive control?**	Molecular: Result in the presence of another tagged protein/gene that marks the organelle of interest. IHC: Result in another model that expresses the marker	Yes/NR/NA If yes add free text to justification.
	**7. Is there an appropriate negative control?**	Molecular: Result in the presence of a tagged protein that does not mark the organelle of interest OR in the absence of a tagged protein (e.g. empty vector, tag only). IHC: Result in absence of marker, AND result in another model than does not express the marker	Yes/NR/NA If yes add free text to justification.
	**Control rating**	Low= no concerns. Unclear/not reported = insufficient data to make a judgement or not reported. High risk = there are concerns	**Low/UNR/High** **If UNR/High add free text to justification**
**Experimental performance, reporting flaws**	**8. Were there sufficient details to judge the performance of molecular experiments?**	Detailed =, allowing repetition of the experiment. Partial = some details, but could not repeat the experiment easily. NR = not reported	D/P/NR/NA
	**9. Did the authors provide evidence that the genetic manipulation did not influence the observed effect?**		Yes/NR/NA If yes add free text to justification.
	**10. Molecular techniques: Additional comments/concerns**		Yes/NR/NA If yes add free text to justification.
	**11. Were there sufficient details to judge the performance of immunochemistry?**	Detailed = allowing easy repetition of the experiment. Partial = some details, but could not repeat the experiment easily. NR = not reported	D/P/NR/NA
	**12. Immunotechniques: Additional comments/concerns**		Yes/NR/NA If yes add free text to justification.
	**13. Type of image analysis.**	Confocal fluorescent/fluorescent NR/light	
	**14. Were there sufficient details to repeat the image analysis?**	Detailed = allowing easy repetition of the experiment. Partial = some details, but could not repeat the experiment easily. NR = not reported	D/P/NR/NA
	**15. Was the optical plane considered?**		Yes/NR/NA
	**16. Additional comments/concerns regarding imaging**		Yes/NR/NA If yes add free text to justification.
	**Experimental performance rating**	Low = no concerns. Unclear/not reported = insufficient data to make a judgement or not reported. High risk = there are concerns	**Low/UNR/High** **If UNR/High add free text to justification**
**Applicability/generalisability**	**Model variability ** **(Did the experiment give the same result in a different model?)**	Yes = low NR = UNR	**Low/UNR** **If low add free text to justification**
**Additional Biases**	**17. Any experimental assumptions?**		Yes/NR/NA If yes add free text to justification.
	**18. Other concerns/** **How was asymmetry measured?**	Was subjective assessment used, if yes the results should be verified independently	Yes/NR/NA If yes add free text to justification.
	**19. Was the marker stated a priori?**	The marker should be stated a priori in the introduction or methods. Or the authors should assess a range of markers clearly stated in the aims. If the authors list the marker in the methods or results only (with no further details or intention) this is unclear/NR.	Yes/NR/No
	**additional rating**	Low= no concerns. Unclear/not reported = insufficient data to make a judgement or not reported. High risk = there are concerns	**Low/UNR/High** **If UNR/High add free text to justification**
**OVERALL RATING**		Low = all domains clearly reported. Unclear = Any domains are unclear, but not high risk. High risk = there is a concern of high risk	**Low/UNR/High**
**JUSTIFICATION**		Free text to explain UNR or High ratings, plus additional free text from signalling questions	

Note that if several overall ratings inform one asymmetry result (if there is an organelle marker and a cell specific marker) then a second overall judgement is made based on the same instructions notes for the overall rating.

**Table A7:** Model Validity Tool Assessments.

	Marker	1.	2.	3.	4.	5.	Marker Validation Rating	6. Positive	7. Negative	Control Rating
***HUVEC (Human Umbilical Vein Endothelial Cells)***										
Wang 2019	PCSK9	PCSK9 released from *HUVEC*	Cytoplasm, Endosome, Lysosome, Cell surface, Endoplasmic reticulum, Golgi apparatus.	Extracellular region, extracellular space, cytoplasm, lysosome, and lysosomal membrane.	**NR**	**NR**	**Low**	NR	Yes	**UNR**
	LDLR	LDLR released from *HUVEC*	Cell membrane, Single-pass type I membrane protein, Membrane, clathrin-coated pit, Golgi apparatus. Endosome, Lysosome.	Lysosome, endosome, and golgi Apparatus.	**NR**	**NR**	**Low**	NR	NR	**High**
	ICAM-1	ICAM-1 released from *HUVEC*	Membrane. Single pass type I membrane protein.	Immunological synapse, extracellular space, plasma membrane, integral component of plasma membrane, and focal adhesion	NR	NR	**Low**	NR	Yes	**UNR**
	VCAM	VCAM-1 released from *HUVEC*	Membrane. Single pass type I membrane protein.	Podosome, extracellular space, early endosome, endoplasmic reticulum, and golgi apparatus.	NR	NR	**Low**	NR	Yes	**UNR**
	SREBP2	SREBP2 released from *HUVEC*	Endoplasmic reticulum membrane. Multi-pass membrane protein. Golgi apparatus membrane. Multi-pass membrane protein. Cytoplasmic vesicle, COPII-coated vesicle membrane. Multi-pass membrane protein.	Golgi membrane, chromarin, nucleus, nucleoplasm, and cytoplasm.	NR	NR	**Low**	NR	Yes	**UNR**
	IL-1α	IL-1α released from *HUVEC*	Cytoplasm.	Extracellular region, extracellular space, cytoplasm, cytosol, and plasma membrane.	NR	NR	**Low**	NR	Yes	**UNR**
	IL-1β	IL-1β released from *HUVEC*	Cytoplasm, cytosol. Lysosome. Secreted, extracellular exosome.	Extracellular region, extracellular space, cytoplasm, lysosome, and cytosol.	NR	NR	**Low**	NR	Yes	**UNR**
	IL-6	IL-6 released from *HUVEC*	Endoplasmic reticulum, Extracellular exosome, cytosol, nucleus.	Extracellular region, extracellular space, endoplasmic reticulum lumen and interleukin 6 receptor complex.	NR	NR	**Low**	NR	Yes	**UNR**
	MCP-1	MCP-1 released from *HUVEC*	-	-	NR	NR	**UNR**	NR	Yes	**UNR**
	CXCL-1	CXCL-1 released from *HUVEC*	Extracellular exosome.	Extracellular region, extracellular space, and granule lumen.	NR	NR	**Low**	NR	Yes	**UNR**
	CXCL-2	CXCL-2 released from *HUVEC*	-	-	NR	NR	**UNR**	NR	Yes	**UNR**
	NOX-4	NOX-4 released from *HUVEC*	Endoplasmic reticulum membrane.	Nucleus, nucleolus, mitochondria, and endoplasmic reticulum.	NR	NR	**Low**	NR	Yes	**UNR**
	LOX-1	LOX-1 released from *HUVEC*	-	-	NR	NR	**UNR**	NR	Yes	**UNR**
***Huh7 (Human Hepatocytes)***										
Mbikay 2014	PCSK9	PCSK9 released from *Huh7*	Cytoplasm, Endosome, Lysosome, Cell surface, Endoplasmic reticulum, Golgi apparatus.	Extracellular region, extracellular space, cytoplasm, lysosome, and lysosomal membrane.	NR	NR	**Low**	Yes	Yes	**Low**
	LDLR	LDLR released from *Huh7*	Cell membrane, Single-pass type I membrane protein, Membrane, clathrin-coated pit, Golgi apparatus. Endosome, Lysosome.	Lysosome, endosome, and golgi apparatus.	NR	NR	**Low**	Yes	Yes	**Low**
	SREBP2	SREBP2 released from *Huh7*	Endoplasmic reticulum membrane. Multi-pass membrane protein. Golgi apparatus membrane. Multi-pass membrane protein. Cytoplasmic vesicle, COPII-coated vesicle membrane. Multi-pass membrane protein.	Golgi membrane, chromarin, nucleus, nucleoplasm, and cytoplasm.	NR	NR	**Low**	NR	Yes	**UNR**
Wang 2020	PCSK9	PCSK9 released from *Huh7*	Cytoplasm, Endosome, Lysosome, Cell surface, Endoplasmic reticulum, Golgi apparatus.	Extracellular region, extracellular space, cytoplasm, lysosome, and lysosomal membrane.	NR	NR	**Low**	Yes	Yes	**Low**
	LDLR	LDLR released from *Huh7*	Cell membrane, Single-pass type I membrane protein, Membrane, clathrin-coated pit, Golgi apparatus. Endosome, Lysosome.	Lysosome, endosome, and golgi apparatus.	NR	NR	**Low**	Yes	Yes	**Low**
	PPARg	PPARg released from *Huh7*	Nucleus	Chromatin, nucleus, nucleoplasm, cytoplasm, and cytosol.	NR	NR	**Low**	NR	Yes	**UNR**
	FoxO3a	FoxO3a released from *Huh7*	Cytoplasm, cytosol, nucleus, mitochondrion matrix, mitochondrion outer membrane, peripheral membrane protein, and cytoplasmic side.	Chromatin, nucleus, nucleoplasm, cytoplasm, and mitochondria.	NR	NR	**Low**	Yes	Yes	**Low**
***LO2 (hepatocytes)***										
Jing 2019	PCSK9	PCSK9 released from *LO2*	Cytoplasm, Endosome, Lysosome, Cell surface, Endoplasmic reticulum, Golgi apparatus.	Extracellular region, extracellular space, cytoplasm, lysosome, and lysosomal membrane.	NR	NR	**Low**	NR	Yes	**UNR**
	LDLR	LDLR released from *LO2*	Cell membrane, Single-pass type I membrane protein, Membrane, clathrin-coated pit, Golgi apparatus. Endosome, Lysosome.	Lysosome, endosome, and golgi apparatus.	NR	NR	**Low**	NR	Yes	**UNR**
	SREBP 1c	SREBP 1c released from *LO2*	Nucleoplasm, cytosol, and golgi apparatus.	Golgi membrane, chromarin, nucleus, nuclear envelope, and nucleoplasm.	NR	NR	**Low**	NR	Yes	**UNR**
***HepG2 (Human Hepatoma)***										
Aggrey 2019	PCSK9	PCSK9 released from *HepG2*	Cytoplasm, Endosome, Lysosome, Cell surface, Endoplasmic reticulum, Golgi apparatus.	Extracellular region, extracellular space, cytoplasm, lysosome, and lysosomal membrane.	NR	NR	**Low**	NR	Yes	**UNR**
	LDLR	LDLR released from *HepG2*	Cell membrane, Single-pass type I membrane protein, Membrane, clathrin-coated pit, Golgi apparatus. Endosome, Lysosome.	Lysosome, endosome, and golgi apparatus.	NR	NR	**Low**	NR	Yes	**UNR**
Ahn 2019	PCSK9	PCSK9 released from *HepG2*	Cytoplasm, Endosome, Lysosome, Cell surface, Endoplasmic reticulum, Golgi apparatus.	Extracellular region, extracellular space, cytoplasm, lysosome, and lysosomal membrane.	NR	NR	**Low**	NR	Yes	**UNR**
Cameron 2008	PCSK9	PCSK9 released from *HepG2*	Cytoplasm, Endosome, Lysosome, Cell surface, Endoplasmic reticulum, Golgi apparatus.	Extracellular region, extracellular space, cytoplasm, lysosome, and lysosomal membrane.	NR	NR	**Low**	Yes	Yes	**Low**
	LDLR	LDLR released from *HepG2*	Cell membrane, Single-pass type I membrane protein, Membrane, clathrin-coated pit, Golgi apparatus. Endosome, Lysosome.	Lysosome, endosome, and golgi apparatus.	NR	NR	**Low**	Yes	Yes	**Low**
Chae 2018	PCSK9	PCSK9 released from *HepG2*	Cytoplasm, Endosome, Lysosome, Cell surface, Endoplasmic reticulum, Golgi apparatus.	Extracellular region, extracellular space, cytoplasm, lysosome, and lysosomal membrane.	NR	NR	**Low**	Yes	Yes	**Low**
	LDLR	LDLR released from *HepG2*	Cell membrane, Single-pass type I membrane protein, Membrane, clathrin-coated pit, Golgi apparatus. Endosome, Lysosome.	Lysosome, endosome, and golgi apparatus.	NR	NR	**Low**	Yes	Yes	**Low**
Chen 2016	PCSK9	PCSK9 released from *HepG2*	Cytoplasm, Endosome, Lysosome, Cell surface, Endoplasmic reticulum, Golgi apparatus.	Extracellular region, extracellular space, cytoplasm, lysosome, and lysosomal membrane.	NR	NR	**Low**	NR	Yes	**UNR**
	LDLR	LDLR released from *HepG2*	Cell membrane, Single-pass type I membrane protein, Membrane, clathrin-coated pit, Golgi apparatus. Endosome, Lysosome.	Lysosome, endosome, and golgi apparatus.	NR	NR	**Low**	NR	Yes	**UNR**
Choi 2017	PCSK9	PCSK9 released from *HepG2*	Cytoplasm, Endosome, Lysosome, Cell surface, Endoplasmic reticulum, Golgi apparatus.	Extracellular region, extracellular space, cytoplasm, lysosome, and lysosomal membrane.	NR	NR	**Low**	Yes	Yes	**Low**
	LDLR	LDLR released from *HepG2*	Cell membrane, Single-pass type I membrane protein, Membrane, clathrin-coated pit, Golgi apparatus. Endosome, Lysosome.	Lysosome, endosome, and golgi apparatus.	NR	NR	**Low**	NR	Yes	**UNR**
	SREBP2	SREBP2 released from *Huh7*	Endoplasmic reticulum membrane. Multi-pass membrane protein. Golgi apparatus membrane. Multi-pass membrane protein. Cytoplasmic vesicle, COPII-coated vesicle membrane. Multi-pass membrane protein.	Golgi membrane, chromarin, nucleus, nucleoplasm, and cytoplasm.	NR	NR	**Low**	NR	Yes	**UNR**
	HNF1α	HNF1α released from *HepG2*	Nucleus	Chromatin, nucleus, transcription regulator complex, and cytoplasm.	NR	NR	**Low**	NR	Yes	**UNR**
Dong 2019	PCSK9	PCSK9 released from *HepG2*	Cytoplasm, Endosome, Lysosome, Cell surface, Endoplasmic reticulum, Golgi apparatus.	Extracellular region, extracellular space, cytoplasm, lysosome, and lysosomal membrane.	NR	NR	**Low**	NR	Yes	**UNR**
Fan 2021	PCSK9	PCSK9 released from *HepG2*	Cytoplasm, Endosome, Lysosome, Cell surface, Endoplasmic reticulum, Golgi apparatus.	Extracellular region, extracellular space, cytoplasm, lysosome, and lysosomal membrane.	NR	NR	**Low**	NR	Yes	**UNR**
	LDLR	LDLR released from *HepG2*	Cell membrane, Single-pass type I membrane protein, Membrane, clathrin-coated pit, Golgi apparatus. Endosome, Lysosome.	Lysosome, endosome, and golgi apparatus.	NR	NR	**Low**	NR	Yes	**UNR**
Gao 2018	PCSK9	PCSK9 released from *HepG2*	Cytoplasm, Endosome, Lysosome, Cell surface, Endoplasmic reticulum, Golgi apparatus.	Extracellular region, extracellular space, cytoplasm, lysosome, and lysosomal membrane.	NR	NR	**Low**	Yes	Yes	**Low**
	LDLR	LDLR released from *HepG2*	Cell membrane, Single-pass type I membrane protein, Membrane, clathrin-coated pit, Golgi apparatus. Endosome, Lysosome.	Lysosome, endosome, and golgi apparatus.	NR	NR	**Low**	Yes	Yes	**Low**
	SREBP2	SREBP2 released from *HepG2*	Endoplasmic reticulum membrane. Multi-pass membrane protein. Golgi apparatus membrane. Multi-pass membrane protein. Cytoplasmic vesicle, COPII-coated vesicle membrane. Multi-pass membrane protein.	Golgi membrane, chromarin, nucleus, nucleoplasm, and cytoplasm.	NR	NR	**Low**	NR	Yes	**UNR**
	HNF1α	HNF1α released from *HepG2*	Nucleus	Chromatin, nucleus, transcription regulator complex, and cytoplasm.	NR	NR	**Low**	NR	Yes	**UNR**
	FoxO3a	FoxO3a released from *Huh7*	Cytoplasm, cytosol, nucleus, mitochondrion matrix, mitochondrion outer membrane, peripheral membrane protein, and cytoplasmic side.	Chromatin, nucleus, nucleoplasm, cytoplasm, and mitochondria.	NR	NR	**Low**	NR	Yes	**UNR**
Fu 2020	PCSK9	PCSK9 released from *HepG2*	Cytoplasm, Endosome, Lysosome, Cell surface, Endoplasmic reticulum, Golgi apparatus.	Extracellular region, extracellular space, cytoplasm, lysosome, and lysosomal membrane.	NR	NR	**Low**	NR	Yes	**UNR**
	LDLR	LDLR released from *HepG2*	Cell membrane, Single-pass type I membrane protein, Membrane, clathrin-coated pit, Golgi apparatus. Endosome, Lysosome.	Lysosome, endosome, and golgi apparatus.	NR	NR	**Low**	NR	Yes	**UNR**
Gu 2017	PCSK9	PCSK9 released from *HepG2*	Cytoplasm, Endosome, Lysosome, Cell surface, Endoplasmic reticulum, Golgi apparatus.	Extracellular region, extracellular space, cytoplasm, lysosome, and lysosomal membrane.	NR	NR	**Low**	NR	Yes	**UNR**
	LDLR	LDLR released from *HepG2*	Cell membrane, Single-pass type I membrane protein, Membrane, clathrin-coated pit, Golgi apparatus. Endosome, Lysosome.	Lysosome, endosome, and golgi apparatus.	NR	NR	**Low**	NR	Yes	**UNR**
	HNF1α	HNF1α released from *HepG2*	Nucleus	Chromatin, nucleus, transcription regulator complex, and cytoplasm.	NR	NR	**Low**	NR	Yes	**UNR**
	SREBP2	SREBP2 released from *HepG2*	Endoplasmic reticulum membrane. Multi-pass membrane protein. Golgi apparatus membrane. Multi-pass membrane protein. Cytoplasmic vesicle, COPII-coated vesicle membrane. Multi-pass membrane protein.	Golgi membrane, chromarin, nucleus, nucleoplasm, and cytoplasm.	NR	NR	**Low**	NR	Yes	**UNR**
Hwang 2020	PCSK9	PCSK9 released from *HepG2*	Cytoplasm, Endosome, Lysosome, Cell surface, Endoplasmic reticulum, Golgi apparatus.	Extracellular region, extracellular space, cytoplasm, lysosome, and lysosomal membrane.	NR	NR	**Low**	NR	Yes	**UNR**
	LDLR	LDLR released from *HepG2*	Cell membrane, Single-pass type I membrane protein, Membrane, clathrin-coated pit, Golgi apparatus. Endosome, Lysosome.	Lysosome, endosome, and golgi apparatus.	NR	NR	**Low**	NR	Yes	**UNR**
	HNF1α	HNF1α released from *HepG2*	Nucleus	Chromatin, nucleus, transcription regulator complex, and cytoplasm.	NR	NR	**Low**	NR	Yes	**UNR**
	SREBP2	SREBP2 released from *HepG2*	Endoplasmic reticulum membrane. Multi-pass membrane protein. Golgi apparatus membrane. Multi-pass membrane protein. Cytoplasmic vesicle, COPII-coated vesicle membrane. Multi-pass membrane protein.	Golgi membrane, chromarin, nucleus, nucleoplasm, and cytoplasm.	NR	NR	**Low**	NR	Yes	**UNR**
	HMGCR	S HMGCR released from *HepG2*	Endoplasmic reticulum membrane. Multi-pass membrane protein. Peroxisome membrane. Multi-pass membrane protein.	Peroxisome, peroxisomal membrane, endoplasmic reticulum, endoplasmic reticulum membrane, and membrane.	NR	NR	**Low**	NR	Yes	**UNR**
Hwang 2021	PCSK9	PCSK9 released from *HepG2*	Cytoplasm, Endosome, Lysosome, Cell surface, Endoplasmic reticulum, Golgi apparatus.	Extracellular region, extracellular space, cytoplasm, lysosome, and lysosomal membrane.	NR	NR	**Low**	Yes	Yes	**Low**
	LDLR	LDLR released from *HepG2*	Cell membrane, Single-pass type I membrane protein, Membrane, clathrin-coated pit, Golgi apparatus. Endosome, Lysosome.	Lysosome, endosome, and golgi apparatus.	NR	NR	**Low**	Yes	Yes	**Low**
	HNF1α	HNF1α released from *HepG2*	Nucleus	Chromatin, nucleus, transcription regulator complex, and cytoplasm.	NR	NR	**Low**	Yes	Yes	**Low**
	SREBP2	SREBP2 released from *HepG2*	Endoplasmic reticulum membrane. Multi-pass membrane protein. Golgi apparatus membrane. Multi-pass membrane protein. Cytoplasmic vesicle, COPII-coated vesicle membrane. Multi-pass membrane protein.	Golgi membrane, chromarin, nucleus, nucleoplasm, and cytoplasm.	NR	NR	**Low**	Yes	Yes	**Low**
Kim 2020	PCSK9	PCSK9 released from *HepG2*	Cytoplasm, Endosome, Lysosome, Cell surface, Endoplasmic reticulum, Golgi apparatus.	Extracellular region, extracellular space, cytoplasm, lysosome, and lysosomal membrane.	NR	NR	**Low**	Yes	Yes	**Low**
	LDLR	LDLR released from *HepG2*	Cell membrane, Single-pass type I membrane protein, Membrane, clathrin-coated pit, Golgi apparatus. Endosome, Lysosome.	Lysosome, endosome, and golgi apparatus.	NR	NR	**Low**	Yes	Yes	**Low**
	HNF1α	HNF1α released from *HepG2*	Nucleus	Chromatin, nucleus, transcription regulator complex, and cytoplasm.	NR	NR	**Low**	NR	Yes	**UNR**
	SREBP2	SREBP2 released from *HepG2*	Endoplasmic reticulum membrane. Multi-pass membrane protein. Golgi apparatus membrane. Multi-pass membrane protein. Cytoplasmic vesicle, COPII-coated vesicle membrane. Multi-pass membrane protein.	Golgi membrane, chromarin, nucleus, nucleoplasm, and cytoplasm.	NR	NR	**Low**	NR	Yes	**UNR**
Lammi 2019	PCSK9	PCSK9 released from *HepG2*	Cytoplasm, Endosome, Lysosome, Cell surface, Endoplasmic reticulum, Golgi apparatus.	Extracellular region, extracellular space, cytoplasm, lysosome, and lysosomal membrane.	NR	NR	**Low**	NR	Yes	**UNR**
	LDLR	LDLR released from *HepG2*	Cell membrane, Single-pass type I membrane protein, Membrane, clathrin-coated pit, Golgi apparatus. Endosome, Lysosome.	Lysosome, endosome, and golgi apparatus.	NR	NR	**Low**	NR	Yes	**UNR**
	HNF1α	HNF1α released from *HepG2*	Nucleus	Chromatin, nucleus, transcription regulator complex, and cytoplasm.	NR	NR	**Low**	NR	Yes	**UNR**
Li 2020	PCSK9	PCSK9 released from *HepG2*	Cytoplasm, Endosome, Lysosome, Cell surface, Endoplasmic reticulum, Golgi apparatus.	Extracellular region, extracellular space, cytoplasm, lysosome, and lysosomal membrane.	NR	NR	**Low**	NR	Yes	**UNR**
	LDLR	LDLR released from *HepG2*	Cell membrane, Single-pass type I membrane protein, Membrane, clathrin-coated pit, Golgi apparatus. Endosome, Lysosome.	Lysosome, endosome, and golgi apparatus.	NR	NR	**Low**	Yes	Yes	**Low**
	SREBP2	SREBP2 released from *HepG2*	Endoplasmic reticulum membrane. Multi-pass membrane protein. Golgi apparatus membrane. Multi-pass membrane protein. Cytoplasmic vesicle, COPII-coated vesicle membrane. Multi-pass membrane protein.	Golgi membrane, chromarin, nucleus, nucleoplasm, and cytoplasm.	NR	NR	**Low**	NR	Yes	**UNR**
	HNF1α	HNF1α released from *HepG2*	Nucleus	Chromatin, nucleus, transcription regulator complex, and cytoplasm.	NR	NR	**Low**	Yes	Yes	**Low**
Masagalli 2021	PCSK9	PCSK9 released from *HepG2*	Cytoplasm, Endosome, Lysosome, Cell surface, Endoplasmic reticulum, Golgi apparatus.	Extracellular region, extracellular space, cytoplasm, lysosome, and lysosomal membrane.	NR	NR	**Low**	NR	Yes	**UNR**
Pel 2020	PCSK9	PCSK9 released from *HepG2*	Cytoplasm, Endosome, Lysosome, Cell surface, Endoplasmic reticulum, Golgi apparatus.	Extracellular region, extracellular space, cytoplasm, lysosome, and lysosomal membrane.	NR	NR	**Low**	NR	Yes	**UNR**
	LDLR	LDLR released from *HepG2*	Cell membrane, Single-pass type I membrane protein, Membrane, clathrin-coated pit, Golgi apparatus. Endosome, Lysosome.	Lysosome, endosome, and golgi apparatus.	NR	NR	**Low**	NR	Yes	**UNR**
	HNF1α	HNF1α released from *HepG2*	Nucleus	Chromatin, nucleus, transcription regulator complex, and cytoplasm.	NR	NR	**Low**	NR	Yes	**UNR**
Pel 2017	PCSK9	PCSK9 released from *HepG2*	Cytoplasm, Endosome, Lysosome, Cell surface, Endoplasmic reticulum, Golgi apparatus.	Extracellular region, extracellular space, cytoplasm, lysosome, and lysosomal membrane.	NR	NR	**Low**	Yes	Yes	**Low**
Weng 2021	PCSK9	PCSK9 released from *HepG2*	Cytoplasm, Endosome, Lysosome, Cell surface, Endoplasmic reticulum, Golgi apparatus.	Extracellular region, extracellular space, cytoplasm, lysosome, and lysosomal membrane.	NR	NR	**Low**	Yes	Yes	**Low**
Wang 2021	PCSK9	PCSK9 released from *HepG2*	Cytoplasm, Endosome, Lysosome, Cell surface, Endoplasmic reticulum, Golgi apparatus.	Extracellular region, extracellular space, cytoplasm, lysosome, and lysosomal membrane.	NR	NR	**Low**	NR	Yes	**UNR**
	LDLR	LDLR released from *HepG2*	Cell membrane, Single-pass type I membrane protein, Membrane, clathrin-coated pit, Golgi apparatus. Endosome, Lysosome.	Lysosome, endosome, and golgi apparatus.	NR	NR	**Low**	NR	Yes	**UNR**
	SREBP2	SREBP2 released from *HepG2*	Endoplasmic reticulum membrane. Multi-pass membrane protein. Golgi apparatus membrane. Multi-pass membrane protein. Cytoplasmic vesicle, COPII-coated vesicle membrane. Multi-pass membrane protein.	Golgi membrane, chromarin, nucleus, nucleoplasm, and cytoplasm.	NR	NR	**Low**	NR	Yes	**UNR**
Wu 2019	PCSK9	PCSK9 released from *HepG2*	Cytoplasm, Endosome, Lysosome, Cell surface, Endoplasmic reticulum, Golgi apparatus.	Extracellular region, extracellular space, cytoplasm, lysosome, and lysosomal membrane.	NR	NR	**Low**	Yes	Yes	**Low**
	LDLR	LDLR released from *HepG2*	Cell membrane, Single-pass type I membrane protein, Membrane, clathrin-coated pit, Golgi apparatus. Endosome, Lysosome.	Lysosome, endosome, and golgi apparatus.	NR	NR	**Low**	Yes	Yes	**Low**
Wu 2021	PCSK9	PCSK9 released from *HepG2*	Cytoplasm, Endosome, Lysosome, Cell surface, Endoplasmic reticulum, Golgi apparatus.	Extracellular region, extracellular space, cytoplasm, lysosome, and lysosomal membrane.	NR	NR	**Low**	NR	Yes	**UNR**
	LDLR	LDLR released from *HepG2*	Cell membrane, Single-pass type I membrane protein, Membrane, clathrin-coated pit, Golgi apparatus. Endosome, Lysosome.	Lysosome, endosome, and golgi apparatus.	NR	NR	**Low**	NR	Yes	**UNR**
	SREBP2	SREBP2 released from *HepG2*	Endoplasmic reticulum membrane. Multi-pass membrane protein. Golgi apparatus membrane. Multi-pass membrane protein. Cytoplasmic vesicle, COPII-coated vesicle membrane. Multi-pass membrane protein.	Golgi membrane, chromarin, nucleus, nucleoplasm, and cytoplasm.	NR	NR	**Low**	NR	Yes	**UNR**
	HMGCR	S HMGCR released from *HepG2*	Endoplasmic reticulum membrane. Multi-pass membrane protein. Peroxisome membrane. Multi-pass membrane protein.	Peroxisome, peroxisomal membrane, endoplasmic reticulum, endoplasmic reticulum membrane, and membrane.	NR	NR	**Low**	NR	Yes	**UNR**
	HNF1α	HNF1α released from *HepG2*	Nucleus	Chromatin, nucleus, transcription regulator complex, and cytoplasm.	NR	NR	**Low**	NR	Yes	**UNR**
Yang 2018	PCSK9	PCSK9 released from *HepG2*	Cytoplasm, Endosome, Lysosome, Cell surface, Endoplasmic reticulum, Golgi apparatus.	Extracellular region, extracellular space, cytoplasm, lysosome, and lysosomal membrane.	NR	NR	**Low**	NR	Yes	**UNR**
	HNF1α	HNF1α released from *HepG2*	Nucleus	Chromatin, nucleus, transcription regulator complex, and cytoplasm.	NR	NR	**Low**	NR	Yes	**UNR**
Yang 2018	PCSK9	PCSK9 released from *HepG2*	Cytoplasm, Endosome, Lysosome, Cell surface, Endoplasmic reticulum, Golgi apparatus.	Extracellular region, extracellular space, cytoplasm, lysosome, and lysosomal membrane.	NR	NR	**Low**	NR	Yes	**UNR**
	SREBP2	SREBP2 released from *HepG2*	Endoplasmic reticulum membrane. Multi-pass membrane protein. Golgi apparatus membrane. Multi-pass membrane protein. Cytoplasmic vesicle, COPII-coated vesicle membrane. Multi-pass membrane protein.	Golgi membrane, chromarin, nucleus, nucleoplasm, and cytoplasm.	NR	NR	**Low**	NR	Yes	**UNR**
	HNF1α	HNF1α released from *HepG2*	Nucleus	Chromatin, nucleus, transcription regulator complex, and cytoplasm.	NR	NR	**Low**	NR	Yes	**UNR**
	FoxO3a	FoxO3a released from *Huh7*	Cytoplasm, cytosol, nucleus, mitochondrion matrix, mitochondrion outer membrane, peripheral membrane protein, and cytoplasmic side.	Chromatin, nucleus, nucleoplasm, cytoplasm, and mitochondria.	NR	NR	**Low**	NR	Yes	**UNR**
Lupo 2019	PCSK9	PCSK9 released from *HepG2*	Cytoplasm, Endosome, Lysosome, Cell surface, Endoplasmic reticulum, Golgi apparatus.	Extracellular region, extracellular space, cytoplasm, lysosome, and lysosomal membrane.	NR	NR	**Low**	Yes	Yes	**Low**
	LDLR	LDLR released from *HepG2*	Cell membrane, Single-pass type I membrane protein, Membrane, clathrin-coated pit, Golgi apparatus. Endosome, Lysosome.	Lysosome, endosome, and golgi apparatus.	NR	NR	**Low**	Yes	Yes	**Low**
	HMGCR	HMGCR released from *HepG2*	Endoplasmic reticulum membrane. Multi-pass membrane protein. Peroxisome membrane. Multi-pass membrane protein.	Peroxisome, peroxisomal membrane, endoplasmic reticulum, endoplasmic reticulum membrane, and membrane.	NR	NR	**Low**	Yes	Yes	**Low**
	FAS	FAS released from *HepG2*	Cell membrane. Single-pass type I membrane protein. Membrane raft.	Extracellular region, cytosol, plasma membrane, and cell surface,	NR	NR	**Low**	NR	Yes	**UNR**
Wang 2020	PCSK9	PCSK9 released from *HepG2*	Cytoplasm, Endosome, Lysosome, Cell surface, Endoplasmic reticulum, Golgi apparatus.	Extracellular region, extracellular space, cytoplasm, lysosome, and lysosomal membrane.	NR	NR	**Low**	Yes	Yes	**Low**
	LDLR	LDLR released from *HepG2*	Cell membrane, Single-pass type I membrane protein, Membrane, clathrin-coated pit, Golgi apparatus. Endosome, Lysosome.	Lysosome, endosome, and golgi apparatus.	NR	NR	**Low**	Yes	Yes	**Low**
	PPARg	PPARg released from *Huh7*	Nucleus	Chromatin, nucleus, nucleoplasm, cytoplasm, and cytosol.	NR	NR	**Low**	NR	Yes	**UNR**
	FoxO3a	FoxO3a released from *Huh7*	Cytoplasm, cytosol, nucleus, mitochondrion matrix, mitochondrion outer membrane, peripheral membrane protein, and cytoplasmic side.	Chromatin, nucleus, nucleoplasm, cytoplasm, and mitochondria.	NR	NR	**Low**	Yes	Yes	**Low**
***JLM3 (hepatocellular carcinoma cells)***										
He 2017	PCSK9	PCSK9 released from *HepG2*	Cytoplasm, Endosome, Lysosome, Cell surface, Endoplasmic reticulum, Golgi apparatus.	Extracellular region, extracellular space, cytoplasm, lysosome, and lysosomal membrane.	NR	NR	**Low**	Yes	Yes	**Low**
	LDLR	LDLR released from *HepG2*	Cell membrane, Single-pass type I membrane protein, Membrane, clathrin-coated pit, Golgi apparatus. Endosome, Lysosome.	Lysosome, endosome, and golgi apparatus.	NR	NR	**Low**	Yes	Yes	**Low**

## References

[1] Collis A., Ross J., Lang S.H. (2017). A systematic review of the asymmetric inheritance of cellular organelles in eukaryotes: A critique of basic science validity and imprecision. PLoS ONE.

[2] Batista N.T., Rebersek M., Vernier P.T., Mali B., Miklavcic D. (2016). Effects of high voltage nanosecond electric pulses on eucaryotic cells (in vitro): A systematic review. Bioelectrochemistry.

[3] Pavan L.M., Rego D.F., Elias S.T., De Luca Canto G., Guerra E.N. (2015). In vitro Anti-Tumor Effects of Statins on Head and Neck Squamous Cell Carcinoma: A Systematic Review. PLoS ONE.

[4] Bus P., Siersema P.D., Van Baal J.W.P.M. (2012). Cell culture models for studying the development of Barrett’s esophagus: A systematic review. Cell. Oncol..

[5] De Vries R.B., Wever K.E., Avey M.T., Stephens M.L., Sena E.S., Leenaars M. (2014). The usefulness of systematic reviews of animal experiments for the design of preclinical and clinical studies. ILAR J..

[6] Sabatine M.S., Giugliano R.P., Keech A.C., Honarpour N., Wiviott S.D., Murphy S.A. (2017). Evolocumab and clinical outcomes in patients with cardiovascular disease. N. Engl. J. Med..

[7] Ballantyne C.M., Neutel J., Cropp A., Duggan W., Wang E.Q., Plowchalk D., Sweeney K., Kaila N., Vincent J., Bays H. (2015). Results of bococizumab, a monoclonal antibody against proprotein convertase subtilisin/Kexin type 9, from a randomized, placebo-controlled, dose-ranging study in statin-treated subjects with hypercholesterolemia. Am. J. Cardiol..

[8] Kereiakes D.J., Robinson J.G., Cannon C.P., Lorezento C., Pordy R., Chaudari U., Colhoun H.M. (2015). Efficacy and safety of the PCSK9 inhibitor alirocumab among high cardiovascular risk patients on maximally tolerated statin therapy: The ODYSSEY COMBO I study. Am. Heart J..

[9] Seidah N.G., Benjannet S., Wickham L., Marcinkiewicz J., Jasmin S.B., Stifani S. (2003). The secretory proprotein convertase neural apoptosis-regulated convertase 1 (NARC-1): Liver regeneration and neuronal differentiation. Proc. Natl. Acad. Sci. USA.

[10] Soutar A.K., Naoumova R.P. (2007). Mechanisms of disease: Genetic causes of familial hypercholesterolemia. Nat. Clin. Pract. Cardiovasc. Med..

[11] Seidah N.G., Awan Z., Chretien M., Mbikay M. (2014). PCSK9: A key modulator of cardiovascular health. Circ. Res..

[12] Rashid S., Curtis D.E., Garuti R., Anderson N.N., Bashmakov Y., Ho Y.K., Hammer R.E., Moon Y.A., Horton J.D. (2005). Decreased plasma cholesterol and hypersensitivity to statins in mice lacking Pcsk9. Proc. Natl. Acad. Sci. USA.

[13] Zhao Z., Tuakli-Wosornu Y., Lagace T.A., Kinch L., Grishin N.V., Horton J.D., Cohen J.C., Hobbs H.H. (2006). Molecular characterization of loss-of-function mutations in PCSK9 and identification of a compound heterozygote. Am. J. Hum. Genet..

[14] Robinson J.G., Farnier M., Krempf M., Bergeron J., Luc G., Averna M., Stroes E.S., Langslet G., Raal F.J., Shahawy M. (2015). Efficacy and safety of alirocumab in reducing lipids and cardiovascular events. N. Engl. J. Med..

[15] Raal F.J., Honarpour N., Blom D.J. (2015). Inhibition of PCSK9 with evolocumab in homozygous familial hypercholesterolaemia (TESLA Part B): A randomised, double-blind, placebo-controlled trial. Lancet.

[16] Shapiro M.D., Fazio S. (2017). PCSK9 and atherosclerosis-lipids and beyond. J. Atheroscler. Thromb..

[17] Barale C., Melchionda E., Morotti A., Russo I. (2021). PCSK9 Biology and Its Role in Atherothrombosis. Int. J. Mol. Sci..

[18] Nicholls S.J., Puri R., Anderson T., Ballantyne C.M., Cho L., Kastelein J.J. (2016). Effect of evolocumab on progression of coronary disease in statin-treated patients: The GLAGOV randomised clinical trial. JAMA.

[19] Adorni M.P., Zimetti F., Lupo M.G., Ruscica M., Ferri N. (2020). Naturally Occurring PCSK9 Inhibitors. Nutrients.

[20] Mohd Ariff A., Abu Bakar N.A., Abd Muid S., Omar E., Ismail N.H., Ali A.M., Mohd Kasim N.A., Mohd Nawawi H. (2020). Ficus deltoidea suppresses endothelial activation, inflammation, monocytes adhesion and oxidative stress via NF-κB and eNOS pathways in stimulated human coronary artery endothelial cells. BMC Complement. Med Ther..

[21] Chumachenko P., Leusova Y.Y., Kheimets G., Chasova E. (2016). Adhesion molecules and subpopulation of mononuclear cells in pulmonary arteries. Kardiologiia.

[22] Khodabandehlou K., Masehi-Lano J.J., Poon C., Wang J., Chung E.J. (2017). Targeting cell adhesion molecules with nanoparticles using in vivo and flow-based in vitro models of atherosclerosis. Exp. Biol. Med..

[23] Higgins J.P.T., Green S. (2011). Cochrane Handbook for Systematic Reviews of Interventions.

[24] Page M.J., McKenzie J.E., Bossuyt P.M., Boutron I., Hoffmann T.C., Mulrow C.D., Shamseer L., Tetzlaff J.M., Akl E.A., Brennan S.E. (2021). The PRISMA 2020 statement: An updated guideline for reporting systematic reviews. BMJ.

[25] Abifadel M., Varret M., Rabes J.P., Allard D., Ouguerram K., Devillers M., Cruaud C., Benjannet S., Wickham L., Erlich D. (2003). Mutations in PCSK9 cause autosomal dominant hypercholesterolemia. Nat. Genet..

[26] Hooijmans C.R., Rovers M.M., de Vries R.B., Leenaars M., Ritskes-Hoitinga M., Langendam M.W. (2014). SYRCLE’s risk of bias tool for animal studies. BMC Med. Res. Methodol..

[27] Wang G., Liu Z., Li M., Li Y., Alvi S.S., Ansari I.A., Khan M.S. (2019). Ginkgolide B Mediated Alleviation of Inflammatory Cascades and Altered Lipid Metabolism in HUVECs via Targeting PCSK-9 Expression and Functionality. BioMed Res. Int..

[28] Mbikay M., Sirois F., Simoes S., Mayne J., Chretien M. (2014). Quercetin-3-glucoside increases low-density lipoprotein receptor (LDLR) expression, attenuates proprotein convertase subtilisin/kexin 9 (PCSK9) secretion and stimulates LDL uptake by Huh7 human hepatocytes in culture. FEBS Open Bio..

[29] Wang D., Yang X., Chen Y., Gong K., Yu M., Gao Y., Wu X., Hu H., Liao C., Han J. (2020). Ascorbic acid enhances low-density lipoprotein receptor expression by suppressing proprotein convertase subtilisin/kexin 9 expression. J. Biol. Chem..

[30] Jing Y., Hu T., Lin C., Xiong Q., Liu F., Yuan J., Zhao X., Wang R. (2019). Resveratrol downregulates PCSK9 expression and attenuates steatosis through estrogen receptor α-mediated pathway in L02 cells. Eur. J. Pharmacol..

[31] Aggrey M.O., Li H.H., Wang W.Q., Wang Y., Xuan L.J. (2019). Indole alkaloid from Nauclea latifolia promotes LDL uptake in HepG2 cells by inhibiting PCSK9. Phytomedicine.

[32] Ahn J., Kim Y.M., Chae H.S., Choi Y.H., Ahn H.C., Yoo H., Kang M., Kim J., Chin Y.W. (2019). Prenylated Flavonoids from the Roots and Rhizomes of Sophora tonkinensis and Their Effects on the Expression of Inflammatory Mediators and Proprotein Convertase Subtilisin/Kexin Type 9. J. Nat. Prod..

[33] Cameron J., Ranheim T., Kulseth M.A., Leren T.P., Berge K.E. (2008). Berberine decreases PCSK9 expression in HepG2 cells. Atherosclerosis.

[34] Chae H.S., You B.H., Kim D.Y., Lee H., Ko H.W., Ko H.J., Choi Y.H., Choi S.S., Chin Y.W. (2018). Sauchinone controls hepatic cholesterol homeostasis by the negative regulation of PCSK9 transcriptional network. Sci. Rep..

[35] Chen H.C., Chen P.Y., Wu M.J., Tai M.H., Yen J.H. (2016). Tanshinone IIA Modulates Low-Density Lipoprotein Uptake via Down-Regulation of PCSK9 Gene Expression in HepG2 Cells. PLoS ONE.

[36] Choi H.K., Hwang J.T., Nam T.G., Kim S.H., Min D.K., Park S.W., Chung M.Y. (2017). Welsh onion extract inhibits PCSK9 expression contributing to the maintenance of the LDLR level under lipid depletion conditions of HepG2 cells. Food Funct..

[37] Dong Z., Zhang W., Chen S., Liu C. (2019). Silibinin A decreases statin-induced PCSK9 expression in human hepatoblastoma HepG2 cells. Mol. Med. Rep..

[38] Fan T.Y., Yang Y.X., Zeng Q.X., Wang X.L., Wei W., Guo X.X., Zhao L.P., Song D.Q., Wang Y.X., Wang L. (2021). Structure-activity relationship and biological evaluation of berberine derivatives as PCSK9 down-regulating agents. Bioorg. Chem..

[39] Gao W.Y., Chen P.Y., Chen S.F., Wu M.J., Chang H.Y., Yen J.H. (2018). Pinostrobin Inhibits Proprotein Convertase Subtilisin/Kexin-type 9 (PCSK9) Gene Expression through the Modulation of FoxO3a Protein in HepG2 Cells. J. Agric. Food Chem..

[40] Fu W., Gao X.P., Zhang S., Dai Y.P., Zou W.J., Yue L. (2020). M17β-Estradiol Inhibits PCSK9-Mediated LDLR Degradation Through GPER/PLC Activation in HepG2 Cells. Front. Endocrinol..

[41] Gu L., Wang Y., Xu Y., Tian Q., Lei G., Zhao C., Gao Z., Pan Q., Zhao W., Nong L. (2017). Lunasin functionally enhances LDL uptake via inhibiting PCSK9 and enhancing LDLR expression in vitro and in vivo. Oncotarget.

[42] Hwang J.T., Kim H.J., Choi H.K., Park J.H., Chung S., Chung M.Y. (2020). Butein Synergizes with Statin to Upregulate Low-Density Lipoprotein Receptor Through HNF1α-Mediated PCSK9 Inhibition in HepG2 Cells. J. Med. Food..

[43] Hwang J.T., Choi E., Choi H.K., Park J.H., Chung M.Y. (2021). The Cholesterol-Lowering Effect of Capsella Bursa-Pastoris Is Mediated via SREBP2 and HNF-1α-Regulated PCSK9 Inhibition in Obese Mice and HepG2 Cells. Foods.

[44] Kim H.J., Lee J., Chung M.Y., Hong S., Park J.H., Lee S.H., Park S.W., Choi H.K., Hwang J.T. (2020). Piceatannol reduces resistance to statins in hypercholesterolemia by reducing PCSK9 expression through p300 acetyltransferase inhibition. Pharmacol. Res..

[45] Lammi C., Bollati C., Lecca D., Abbracchio M.P., Arnoldi A. (2019). Lupin Peptide T9 (GQEQSHQDEGVIVR) Modulates the Mutant PCSK9D374Y Pathway: *In vitro* Characterization of its Dual Hypocholesterolemic Behavior. Nutrients.

[46] Li H.H., Li J., Zhang X.J., Li J.M., Xi C., Wang W.Q., Lu Y.L., Xuan L.J. (2020). 23,24-Dihydrocucurbitacin B promotes lipid clearance by dual transcriptional regulation of LDLR and PCSK9. Acta Pharmacol. Sin..

[47] Masagalli J.N., BasavanaGowda M.K., Chae H.S., Choi W.J. (2021). Synthesis of Moracin C and Its Derivatives with a 2-arylbenzofuran Motif and Evaluation of Their PCSK9 Inhibitory Effects in HepG2 Cells. Molecules.

[48] Pel P., Chae H.S., Nhoek P., Kim Y.M., Khiev P., Kim G.J., Nam J.W., Choi H., Choi Y.H., Chin Y.W. (2020). A stilbene dimer and flavonoids from the aerial parts of Chromolaena odorata with proprotein convertase subtilisin/kexin type 9 expression inhibitory activity. Bioorg. Chem..

[49] Pel P., Chae H.S., Nhoek P., Kim Y.M., Chin Y.W. (2017). Chemical Constituents with Proprotein Convertase Subtilisin/Kexin Type 9 mRNA Expression Inhibitory Activity from Dried Immature Morus alba Fruits. J. Agric. Food Chem..

[50] Weng X., Lou Y.Y., Wang Y.S., Huang Y.P., Zhang J., Yin Z.Q., Pan K. (2021). New dammarane-type glycosides from Gynostemma pentaphyllum and their lipid-lowering activity. Bioorg. Chem..

[51] Wang J., Wang Y.S., Huang Y.P., Jiang C.H., Gao M., Zheng X., Yin Z.Q., Zhang J. (2021). Gypenoside LVI improves hepatic LDL uptake by decreasing PCSK9 and upregulating LDLR expression. Phytomedicine.

[52] Wu C., Xi C., Tong J., Zhao J., Jiang H., Wang J., Wang Y., Liu H. (2019). Design, synthesis, and biological evaluation of novel tetrahydroprotoberberine derivatives (THPBs) as proprotein convertase subtilisin/kexin type 9 (PCSK9) modulators for the treatment of hyperlipidemia. Acta Pharm. Sin. B.

[53] Wu Y.-R., Li L., Sun X.-C., Wang J., Ma C.-Y., Zhang Y., Qu H.-L., Xu R.-X., Li J.-J. (2020). Diallyl disulfide improves lipid metabolism by inhibiting PCSK9 expression and increasing LDL uptake via PI3K/Akt-SREBP2 pathway in HepG2 cells. Nutr. Metab. Cardiovasc. Dis..

[54] Yang S.H., Xu R.X., Cui C.J., Wang Y., Du Y., Chen Z.G., Yao Y.H., Ma C.Y., Zhu C.G., Guo Y.L. (2018). Liraglutide downregulates hepatic LDL receptor and PCSK9 expression in HepG2 cells and db/db mice through a HNF-1a dependent mechanism. Cardiovasc. Diabetol..

[55] Yang X., Zhang J., Chen L., Wu Q., Yu C. (2018). Chitosan oligosaccharides enhance lipid droplets via down-regulation of PCSK9 gene expression in HepG2 cells. Exp. Cell Res..

[56] Lupo M.G., Macchi C., Marchiano S., Cristofani R., Greco M.F., Dall’Acqua S., Chen H., Sirtori C.R., Corsini A., Ruscica M. (2019). Differential effects of red yeast rice, Berberis aristata and Morus alba extracts on PCSK9 and LDL uptake. Nutr. Metab. Cardiovasc. Dis..

[57] He M., Hou J., Wang L., Zheng M., Fang T., Wang X., Xia J. (2017). Actinidia chinensis Planch root extract inhibits cholesterol metabolism in hepatocellular carcinoma through upregulation of PCSK9. Oncotarget.

[58] Liu Y., Beyer A., Aebersold R. (2016). On the dependency of cellular protein levels on mRNA abundance. Cell.

[59] Csardi G., Franks A., Choi D.S., Airoldi E.M., Drummond D.A. (2015). Accounting for experimental noise reveals that mRNA levels, amplified by post-transcriptional processes, largely determine steady-state protein levels in yeast. PLoS Genet..

[60] Silva G.M., Vogel C. (2016). Quantifying gene expression: The importance of being subtle. Mol. Syst. Biol..

[61] Cheng Z., Teo G., Krueger S., Rock T.M., Koh H.W., Choi H., Vogel C. (2016). Differential dynamics of the mammalian mRNA and protein expression response to misfolding stress. Mol. Syst. Biol..

[62] Garg A., Simha V. (2007). Update on dyslipidemia. J. Clin. Endocrinol. Metab..

[63] Zhang D.W., Lagace T.A., Garuti R., Zhao Z., McDonald M., Horton J.D., Cohen J.C., Hobbs H.H. (2007). Binding of proprotein convertase subtilisin/Kexin type 9 to epidermal growth factor-like repeat a of low-density lipoprotein receptor decreases receptor recycling and increases degradation. J. Biol. Chem..

[64] Jeong H.J., Lee H.S., Kim K.S., Kim Y.K., Yoon D., Park S.W. (2008). Sterol-dependent regulation of proprotein convertase subtilisin/kexin type 9 expression by sterol-regulatory element binding protein-2. J. Lipid Res..

[65] Horton J.D., Cohen J.C., Hobbs H.H. (2007). Molecular biology of PCSK9: Its role in LDL metabolism. Trends Biochem. Sci..

[66] Costet P., Cariou B., Lambert G., Lalanne F., Lardeux B., Jarnoux A.L., Grefhorst A., Staels B., Krempf M. (2006). Hepatic PCSK9 expression is regulated by nutritional status via insulin and sterol regulatory element-binding protein 1c. J. Biol. Chem..

[67] Li H., Dong B., Park S.W., Lee H.S., Chen W., Liu J. (2009). Hepatocyte nuclear factor 1alpha plays a critical role in PCSK9 gene transcription and regulation by the natural hypocholesterolemic compound berberine. J. Biol. Chem..

